# Global Property Prediction: A Benchmark Study on Open-Source, Perovskite-like Datasets

**DOI:** 10.1021/acsomega.1c00991

**Published:** 2021-05-03

**Authors:** Felix Mayr, Alessio Gagliardi

**Affiliations:** Department of Electrical and Computer Engineering, Technical University of Munich, Munich 80333, Germany

## Abstract

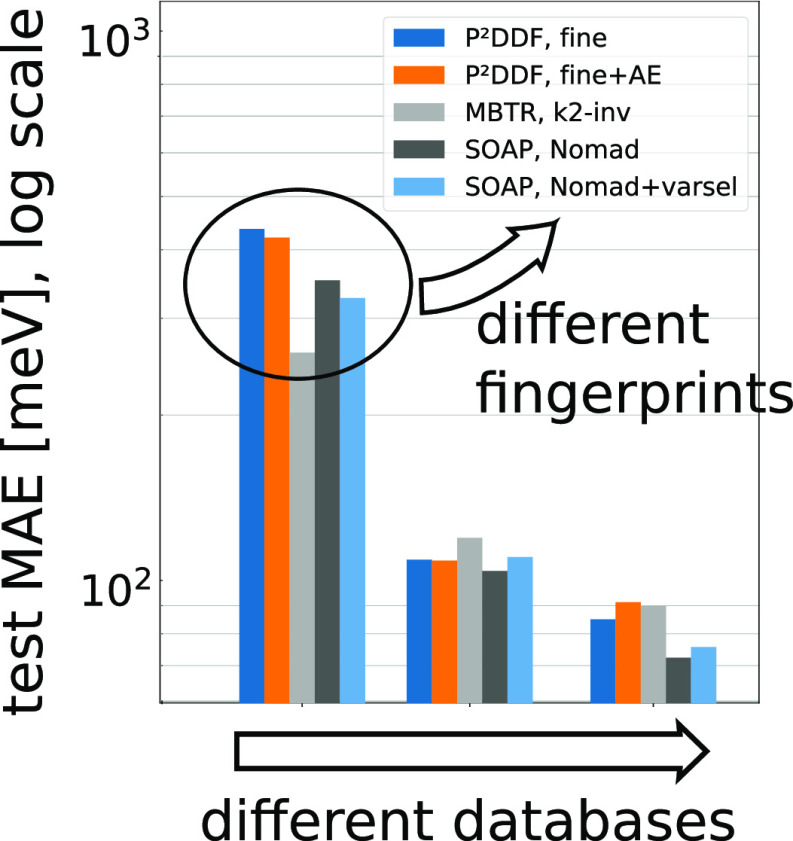

Screening combinatorial space for novel materials, such as perovskite-like ones for photovoltaics, has resulted in a high amount of simulated high-throughput data and analysis thereof. This study proposes a comprehensive comparison of structural fingerprint-based machine learning models on seven open-source databases of perovskite-like materials to predict band gaps and energies. It shows that none of the given methods, including graph neural networks, are able to capture arbitrary databases evenly, while underlining that commonly used metrics are highly database-dependent in typical workflows. In addition, the applicability of variance selection and autoencoders to significantly reduce fingerprint size indicates that models built with common fingerprints only rely on a submanifold of the available fingerprint space.

## Introduction

Perovskite-like materials are of paramount interest in the creation of novel photovoltaic devices. While existing perovskite materials, such as CH_3_NH_3_PbI_3_, are unstable and/or contain toxic lead,^1,2^ the available, combinatorial space of possible candidate compounds is extensive.^3^ This is especially interesting when considering mixtures and different structural phases, which might have widely varying properties.^4,5^ Notably for binary mixtures of selected ions, it is already well established that the relation between an experimentally measured property (e.g., band gap) and material concentrations can be fit with simple, analytic functions.^5,6^ With the industry-led rise of machine learning (ML) methods, there has been growing interest to predict such a relationship in the high-dimensional space of all possible compounds using ML techniques.^7,8^

While these approaches have been used for years in engineering and science in general,^9^ the widespread application in computational materials science is relatively new and accompanied by the (re-)development of a wide range of “fingerprinting functions”.^10−20^ These are necessary to encode the typical atomic and structural information describing materials of interest into a numerical vector format necessary for common ML techniques. Notably even more recently, the usage of graph representations also allows us to skip the explicit fingerprinting step, allowing dynamic learning of numerical representations for atomic neighborhoods from structural graphs.^21−24^ For modeling computationally heavy quantum-chemistry calculations, two major approaches can be discriminated. In the first, one tries to replace certain parts of already established frameworks with ML models, e.g., the parameterization of molecular forcefields^25,26^ or the density functional in density-functional theory (DFT).^27^ The second approach tries to create a surrogate model for prediction of materials properties given only the fingerprints as an input; typical properties for prediction with such a surrogate model are stability/formation energy terms,^12,19,28−31^ band gaps,^7,19,32−37^ or even specific medication properties.^38^ Recent efforts also focus on the prospects of creating “new” materials from generative models or directly feeding the structural graph to a neural-network approximator.^23,39−41^

This study focuses on the surrogate model approach applied to crystalline, perovskite-like materials. In this field, most new methods or supposed performance improvements are only demonstrated with proprietary or novel datasets, severely limiting comparability to preexisting approaches and effectively hindering objective assessment of method performance across the field.^32,36,42^ This is a direct result of the lack—to the author’s knowledge—of a generally accepted, consistently annotated, and high-quality benchmark database for crystalline materials, which could be used for benchmarking of new methods, such as GDB-17 and its offspring QM9 for organic systems.^43,44^ a It should also be noted here that diverse databases—inevitably necessary for a complete surrogate model—tend to generate very large fingerprint vectors, which pose a theoretical and practical problem, when the size of the fingerprint is larger than the number of datapoints available for model building, possibly deteriorating performance.^47^

Most studies seem to implicitly employ both the regularizing properties of ridge regression, as well as the (arbitrary) “metric” induced by a kernel function and do not warrant further attention to this problem.^17,19,36^

**Figure 1:**
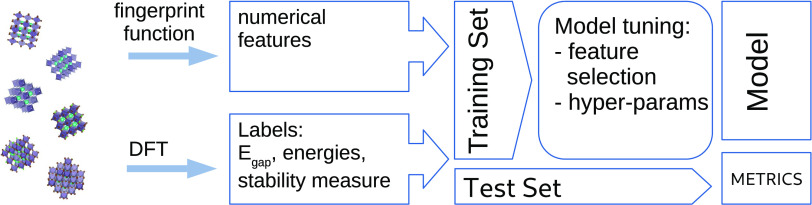
Typical materials prediction workflow for building a surrogate using structural fingerprints. The cost of DFT is typically magnitudes higher than for model evaluation or fingerprinting.

Herein, a typical materials science surrogate modeling approach (compare Figure 1) employing the Kernel ridge regression (KRR) method is used on a variety of preexisting high-throughput databases of various crystalline, perovskite-like materials.^19,36,48−51^ A host of different fingerprinting functions are compared,^13,16,17^ including an improved, competitive version of the property density distribution function (PDDF).^19^ In addition, the graph neural network (GNN) architecture from Xie^23^ is employed as a reference for a competing approach to the problem.

To assess the influence of KRR in squashing the dimensionality of the problem, this study employs a statistical feature selection process using variance thresholding and dimensionality reduction with neural-network autoencoders.^19,52^ It should be noted that this application of the autoencoder is really just for nonlinear dimensionality reduction (similar to PCA), while, for example, studies focusing on molecules have picked up generative models from text processing to create new SMILES-strings—an approach that can not be adopted to crystalline solids, which lack any canonical textual description.^40^

The results underline that actual model accuracy as commonly published depends strongly on the dataset. Intra-dataset even varying methodologies does yield comparable results within the estimated errors for band gap predictions, while no single fingerprinting method is facilitating the creation of equally accurate models for all datasets. Analysis of the fingerprints reveals that models only rely on a subset of the available information in each at the given dataset scales.

## Methods

A typical property-predicting ML surrogate model for materials science is created in a supervised-learning setting on a sufficiently large set of (atomic structure, property)-tuples. The arguably most simple way to do this is to take basic compositional information, such as the fractional occurence of constituents, and then either fit a classic (non)-parametric model or train an artificial neural network (ANN). A natural next step is to use means and higher moments of the property distribution over all atoms in a given structure as a numerical input vector, which yields surprisingly good results although it is completely insensitive to any structural differences.^53^ For a constrained space of the input structures, which follow a given chemical structure (such as the perovskite-like ABX_3_ one), one can also do this in a fine-grained “per-site” way and include basic structural information.^35,37^

Although this could be used for structurally diverse perovskites like those encountered in the work of Kim et al.^51^ as well, it would entail a significant systematic and nonreducible error, as evidenced in Table 2. Countering the argument that the features used therein are far from exhausting the realm of possibilities, any resulting *n*-to-1 mapping still would have an error of the same magnitude as evidenced by calculating the mean absolute error (MAE) across all datapoints to 0.33 eV, assuming all *n* possibilities for each stoichiometry map to the mean band gap of each. While one can certainly try to find a way to incorporate different structural motifs, this runs counter to the designated goal of this study to provide an equal comparison across varying databases.

Thus, the remainder of this study will focus on fingerprints, which allow incorporation of structural information independent of a predetermined system. Notably, this includes the sine matrix,^16^ the Smooth Overlap of Atomic Positions fingerprint (SOAP),^13,45^ the many-body tensor representation (MBTR),^17^ and the property density distribution function (PDDF),^19^ leaving out approaches only commonly employed with molecules and various adapations of local atomic symmetry functions.^10−12,15,18^

Except for the Coulomb-matrix-derived sine matrix,^12^ all employed descriptors are derived from a shared basis, where for a given atom *j*, the environment is described by the atomic density of its neighbors *i* (see refs (13, 45))^b^
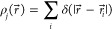
1Also, as this formalism is “atom-centered”, any derived, numerical fingerprint is atom-local first and it is necessary to transform it to a “global” fingerprint to be used for predicting system-total properties for systems of varying compositions. This transformation is done using special kernel functions with kernel-based ML techniques^45^ or by averaging the output over all atoms.^19,46,54^

While the SOAP fingerprint consists of the coefficients for expansion of the atomic density with radial and spherical basis functions, both MBTR and PDDF extend upon classic radial distribution functions. Within the MBTR approach, both partial radial and angular distribution functions can be parameterized on different scales. On the contrary, the PDDF weights contributions to a global RDF with atomic properties. A thorough review of all used methods can be found in the Supporting Information (SI).

A common problem with SOAP and partial RDF-based fingerprints is that they tend to generate large () fingerprint vectors, which—when combined with a nonregularizing regression method—could lead to model overfitting if the dataset is also in the lower  range and require large amounts of computational resources for kernel computation. While sparsity of the individual fingerprints and the regularization part of KRR seem to alleviate this concern in a nonexplicit way in most previous studies, this study employs variance selection to methodologically shrink the input feature vector and observe the influence on surrogate modeling.

Additionally, dimensionality reduction techniques are employed to shrink the fingerprint vector, reducing the risk of overfitting and computational cost as well.^55^ The underlying assumption is that for most small-scale datasets, the structural and compositional variation within certain restrictions (such as “only pervoskite-like” materials) is changing fingerprints in such a way that this change can be projected onto a lower-dimensional manifold.

**Figure 2:**
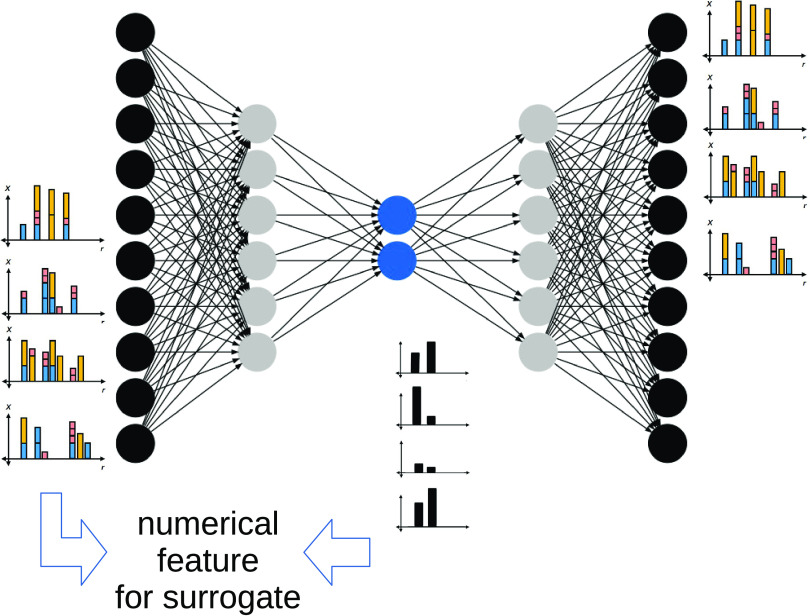
Network architecture of a two-layer autoencoder. The input is a numerical vector, and the output tries to reconstruct the input, passing through a bottleneck, the so-called “latent space” (blue). Surrogate models are then either built on top of the input or the intermediate representation.

For this purpose, this work proposes to use autoencoders, which are an unsupervised learning method using neural networks, passing the fingerprint as an input through an “encoder” network, leading up to a “latent” layer (which is smaller than the original) and then up again through a mirrored network (the “decoder”) such that the original input is recreated (see Figure 2). For building a regression model, the encoder then creates a compressed representation of all of the fingerprints in question, and these are fed to classical Kernel ridge regression. Furthermore, one could—depending on the design of the study—feed all candidate compounds to the autoencoder including the ones, where one would like to do ML-based predictions (because training it is much cheaper than running full DFT for all). Also, the reduced and ideally nonsparse feature vector could allow us to optimize for fingerprints (and subsequent structures) with a desired property within the restricted compositional and structural space of a numerical experiment.^40^

As a contender to these classic approaches, GNNs are nowadays widely considered the state of the art for structural surrogate models. However, they have shown inferior performance at small database sizes,^56^ and even the initial paper from Xie and Grossman^23^ applied on the Materials Project database^57^ was later shown to be performing worse than a classic SOAP approach.^24^ In the context of the classic fingerprinting functions, it is also interesting to note that the graph-convolutional operation could itself be seen as dynamically learning a neighborhood fingerprint per atom.^21^ Notably, this approach also inherits a certain “blindness” to three-dimensional environments as the graph topology only includes two-point distance information. As a comparison point for the fixed fingerprint parameterizations, this study employs the network architecture and graph construction demonstrated by Xie.^23^

## Data

The availability of consistent, high-quality data is crucial for building an ML model and eventual benchmarking of different modeling approaches. Due to the lack of a shared, reproducible benchmark with a lot of common materials properties in the “solid-state” community, authors tend to create their own datasets, when publishing new methods or researching new problems.^32,36,42^ This process introduces the danger of the data being biased in an inadvertented way and thus giving unrealistic, nongeneralizable results. In addition, creating a suitable, high-quality DFT database of crystalline solids is a challenging task itself: one would like to have a high fraction of “physical” systems, e.g., at their minimal energy, which requires extensive structural relaxations or even metadynamics to sample different likely substructures.^51^ Calculations should also use a shared set of sufficiently exact parameters for all calculations to converge, which is hard to achieve with varying cell sizes and some of the proposed inputs exhibiting metallic behavior without human intervention even in advanced computational workflows.^57^ Once a suitable amount of structures is relaxed, it still has to be assured that the model relates to physical reality, e.g., in the case of band gap as a property by incorporating spin–orbit coupling and hybrid DFT, which generally seems to give band gaps in good agreement with experiments compared to the underestimation by generalized gradient approximation (GGA).^58,59^

While creating a high-quality database, taking into account all of these considerations is necessary to create a useful, physically exact surrogate model, and for methodological development, the usage of datasets of lower methodological complexity is definitely possible. Although a Perdew–Burke–Ernzerhof (PBE)-trained model might not yield accurate property values, it can be expected that model accuracy will not get worse than the PBE baseline—which is still used for screening today—when trained on hybrid training data, while possibly leading to a significant performance increase. Thus, herein, the choice fell on existing datasets of varying provenance and methodological backgrounds to assess whether the given methods are able to build effective surrogate models across different databases—a mutual theme is the inclusion of perovskite-like structures.^19,36,48−51^

A large (≈19k samples) dataset of cubic perovskites is used from Castelli et al.^48,60^ It consists of cubic oxide perovskite scaffolds, featuring a wide range of cations and fractional replacement of the oxygen with flour, nitrogen, and sulfur. Optimized cubic structures were found by scanning a range of lattice parameters and relaxing the resulting structure using DFT with the RPBE functional. For all nonmetals, direct and indirect band gaps were subsequently calculated with the GLLB-SC functional, which yields good agreement with experiments. A subselection of these compounds (only with O and N anion) and the same methodology were employed to derive a database of Ruddlesden—Popper layered perovskites.^49^

Compared to these basic databases mainly varying the composition, the “A hybrid organic–inorganic perovskite dataset” by Kim et al.^51^ includes molecular cations A in a “classic” ABX_3_ halide perovskite scaffolds (with B = Ge/Sn/Pb and a halide X). Basic scaffold structures and cells were selected by running a minima-hopping simulation for initial ASnI_3_ compounds, resulting in a large number of different structural motifs, replacing the other sites, and running a structural relaxation with the rPW86 functional. Band gaps were then evaluated with the final structures at the position of both the direct and indirect gaps in the relaxation calculation using hybrid DFT (HSE06).

In addition, a database of A_2_BCX_4_-type materials was selected,^50^ which are similar in size and scope to typical double perovskites. Structures are based on six different prototypes with the composition determined by empirical rules. Structures were optimized using PBEsol, with meta-GGA then used for energy calculations and GLLB-SC for accurate band gaps.

Two smaller databases based on plain GGA and simple relaxation of base structures are also included: first, a recently published dataset based on experimentally available two-dimensional (2D)-perovskite compounds.^36^ The structures therein generally resemble surfaces and thus exhibit widely varying cell sizes^c^. Second, the database used by the authors in the introduction of the PDDF consisting of relaxed, lead-free, inorganic mixed 2 × 1 × 2 cubic cell perovskites calculated at the GGA level using an LCAO approach even for band gaps is included.^19^

As all of these databases incorporate a wide variety of species, fingerprints treating each pair of possible species separately (SOAP, MBTR) might be at a disadvantage and thus the crystalline dataset used in the Nomad-2018-Kaggle-competition consisting of a wide variety of (Al*_x_*Ga*_y_*In_1–*x*–*y*_)_2_O_3_ compounds was included as a further reference.^46^

A basic overview of core properties of all used databases is found in Table 1, including summary statistics over all datapoints for structure size and the type of the band gap/energy property. Note that these properties are not comparable between different databases.

**Table 1:** Overview of the Used Databases^a^

	total compounds	unique species	size	max cell vector [Å]	avg. cell vector [Å]	band gap [eV]
Kim^51^	1346	11	15.1 [9–21]	7.4 [4.4–11.7]	6.0 [4.3–7.6]	3.8 [1.52–6.63] (HSE06)
Pandey^50^	1341	25	19.8 [16–32]	11.9 [7.0–21.5]	7.7 [6.4–10.5]	2.1 [0.01–4.28] (GLLB-SC)
Stanley^19^	344	9	20 [20–20]	11.6 [10.9–13.9]	9.2 [8.7–9.9]	1.4 [0.35–3.08] (LCAO, PBE)
Castelli^49^	1984	47	20.9 [14–44]	10.6 [7.3–24.0]	6.6 [5.5–9.4]	3.5 [0–8.44] (GLLB-SC)
Castelli^48^	18 928 [735 nonzero gaps]	56	5 [5–5]	4.1 [3.3–5.7]	4.1 [3.3–5.7]	0.1 [0–7.90] (GLLB-SC)
Marchenko^36^	445	16	48.8 [4–452]	27.7 [9.4–102.0]	13.2 [6.4–22.9]	2.4 [1.65–3.53] (LCAO, n/a)
Sutton^46^	3000	4 [4–4]	61.7 [10–80]	15.1 [9.0–28.0]	9.0 [4.8–10.8]	2.1 [0.0–5.84] (PBE)

a Size (number of atoms), length of the maximal/geometrical average cell vector (Å), and band gap are all given in the format: “mean [minimum – maximum]”. For the band gap, the chosen exchange functional is given within parentheses.

## ML Experiments

To facilitate the comparison objective, the property prediction workflow is standardized across all databases and no dataset-tailored parameters or methods beyond the statistical model fitting/training procedure are used (see Figure 1). First, each randomly shuffled dataset is split into an 80% set for training and validation, while the remaining 20% is set aside for testing. Then, the chosen fingerprinting (or graphing) function is applied to the structures, either feeding the output to an intermediate step reducing the fingerprint with an autoencoder or variance selection, or directly building the model using the fingerprints (or graph) and a selected global property as a target. For all fingerprint models, 5-fold cross-validation was used to tune hyperparameters of a Kernel ridge regression model using radial basis functions. Finally, the resulting model is evaluated on the test set, resulting in an estimation of prediction accuracy in Table 2 for direct band gaps and for per-atom (formation) energies for each compound (in the SI). Each model is evaluated using the mean absolute error (MAE) metric to estimate the error of the prediction and the *R*^2^-score (coefficient of determination) to classify the adherence to the ideal (prediction = ground truth) relation, as the MAE alone depends strongly on the dataset. The MAE metric was deliberately chosen over the root-mean-square error (RMSE) used in similar works^32,61^ because it de-emphasizes outliers in predictions and is independent of the sample size.^62^ Also for a materials prediction workflow, where the end result will be validated with high-level calculations or experiments from a relatively large array of surrogate-qualified candidates, singular predictions which are off by a large amount are less relevant. The results shown in Table 2 are the average of 10 different train test splits with the standard deviation used as an error estimate. In face of the small datasets and nonstandardized train test splits, this method was chosen to avoid sampling a pathological, nongeneralizable split.^19,61^

**Table 2:** Results for Predicting the Calculated Band Gaps for Different Methods^a^

	Kim^51^	Pandey^50^	Stanley^19^	Castelli^48^	Castelli^49^	Marchenko^36^	Sutton^46^
handpicked	381 ± 11						
dummy	884 ± 34	730 ± 19	323 ± 23	1270 ± 73	1530 ± 46	332 ± 15	845 ± 16
sine matrix, eigenspectrum	368 ± 15	538 ± 39	212 ± 15	1088 ± 77	1102 ± 60	298 ± 22	141 ± 8
GNN, Xie	185 ± 13	154 ± 10	130 ± 18	655 ± 71	262 ± 21	107 ± 9	92 ± 9
PDDF, basic	172 ± 11	199 ± 13	134 ± 11	930 ± 80	551 ± 16	179 ± 11	101 ± 4
PDDF, fine	141 ± 8	139 ± 14	114 ± 12	888 ± 57	481 ± 19	176 ± 20	90 ± 4
PDDF, fine + AE	142 ± 6	143 ± 7	110 ± 12	879 ± 61	490 ± 28	170 ± 19	91 ± 4
P^2^DDF, basic	159 ± 12	172 ± 14	136 ± 19	888 ± 69	521 ± 19	207 ± 32	96 ± 3
P^2^DDF, fine	118 ± 12	116 ± 9	109 ± 7	834 ± 55	436 ± 22	176 ± 29	85 ± 3
P^2^DDF, fine + AE	120 ± 7	113 ± 8	109 ± 6	806 ± 48	421 ± 27	178 ± 31	91 ± 2
MBTR, k2-inv	124 ± 7	159 ± 12	120 ± 11	709 ± 50	260 ± 15	143 ± 11	90 ± 5
MBTR, k2-rdf	128 ± 7	144 ± 13	126 ± 10	786 ± 57	305 ± 18	140 ± 18	93 ± 6
SOAP, Marchenko	100 ± 8	85 ± 9	109 ± 7	1067 ± 75	349 ± 27	494 ± 90	70 ± 5
SOAP, De	107 ± 6	97 ± 9	108 ± 8	1071 ± 74	329 ± 25	442 ± 95	78 ± 4
SOAP, Nomad	106 ± 7	90 ± 8	104 ± 10	926 ± 64	352 ± 27	339 ± 112	72 ± 4
SOAP, Marchenko, LR	110 ± 11	96 ± 7	122 ± 10	1288 ± 130	645 ± 58	939 ± 330	75 ± 3
SOAP, Marchenko + varsel	101 ± 6	111 ± 10	123 ± 8	738 ± 52	309 ± 24	132 ± 17	77 ± 6
SOAP, De + varsel	106 ± 9	112 ± 10	116 ± 7	777 ± 50	339 ± 23	135 ± 20	78 ± 4
SOAP, Nomad + varsel	105 ± 6	114 ± 11	110 ± 9	734 ± 45	327 ± 18	125 ± 18	76 ± 2
SOAP, Marchenko, LR + varsel	99 ± 8	90 ± 5	104 ± 8	745 ± 48	324 ± 27	129 ± 25	76 ± 3

a All results in meV for the mean absolute error (MAE). The parameters for specific identifiers are listed in the Supporting Information. Note here that P^2^DDF is used as a shorthand for the product-weighting proposed in ref (66); varsel indicates that the machine learning was trained on the variance-selected features of the specified fingerprint function.

While nothing precludes the use of neural networks or other regression methods, Kernel ridge regression was used throughout all experiments using fingerprint representations due to its low number of tunable parameters and its popularity within previous work.^17−19,36,46,61^ The “meta” kernel approach was evaluated as well, specifically for the SOAP descriptor, but ultimately discarded, as it requires an enormous amount of computational time for kernel evaluation, while only marginally improving results.^45,54^

Although there is a magnitude of global, macroscopic properties available,^28^ the employed databases only include band gap and energy measures. While the band gap can be used “as is” as a global property and is comparable except for intrinsic differences in the method’s accuracy between databases, energy measures vary, with the availability ranging from bare total DFT energies to formation energies within different, noncomparable frameworks. Remedying this would require recalculating all compounds in a shared framework, which is beyond the scope of this study. Thus, the focus lies on the band gap prediction models, with performance of prediction models for different kinds of formation energies and intensive “per-atom” DFT energies shown in the SI.

To assess a baseline performance level for the more advanced methods, this study includes the results of a dummy regressor, returning the mean of the training dataset for all “predictions” on the test set. Only on the hybrid perovskite database,^51^ some handpicked features (eight features: avg., site-specific properties for the ions^32,53^) were considered and show a relatively good model (*R*^2^ ≈ 0.79) with an MAE of ≈380 meV for the band gap. At this point, it becomes apparent that the MAE alone gives no real indication for the quality of a surrogate model. For example, the dummy regressor on the Marchenko database^36^ achieves “performance” similar to the primitive predictor on the Kim database,^51^ which already improves significantly on the dummy prediction there, both with the MAE and the *R*^2^ score. With the band gap prediction, creation of a decent (*R*^2^ ≃ 1) model for the full dataset of cubic perovskites^48^ was not possible and thus the subset of perovskites with nonzero band gaps was selected for modeling.^32^

For the SOAP fingerprint, the sparse, single-constituent fingerprints of a crystal were taken and averaged to create a global descriptor;^46,54^ readers should take note that the original authors of SOAP publicly endorse^d^ using a “fingerprint-informed” way to create an average for structures, which has not seen broad adoption and thus was not used in this publication.^63,64^ The other parameters used in fingerprint creation e were picked from the existing literature, where widely varying numbers for the modeled cutoff radius and the number of radial and spherical basis functions are given without any reasoning (see the SI for a listing).^13,36,45,46^ Assuming that large systems (such as in refs (36 and 49)) might benefit from modeling a larger cutoff radius around individual atoms, the parameters from ref (36) were also included with a radius of 16 Å.^f^

Kernel calculation for the full fingerprints is very compute-intensive with fingerprint size in the five-digit range depending on the number of species included in the data. Thus individual features were min-max-scaled to the [0, 1]-interval and variance selection with a 0.01 threshold was employed to significantly reduce the fingerprint size before scaling the data to unit variance and feeding the data to a KRR model using radial basis functions. The resulting models match or exceed the performance of the usage of the full fingerprint, where a simple linear kernel and no scaling were used as the radial basis function kernel required more computational time and did not improve accuracy.

For the MBTR, only the k2 part was used, as this already results in a sizable fingerprint of size *s*^2^·*b*, where *s* is the number of species in the database and *b* is the number of discrete bins, used to discretize the fingerprint on the given cutoff radius of 16 Å^g^. *b* = 10 was chosen and worked well with both the partial rdf equivalent representation and discretization over the inverse radius and no scaling applied before feeding the data to the radial basis KRR model. Using both the full MBTR including the angular parts and setting *b* = 100 for the k2 version^17,61^ did not produce improved results consistently and significantly increased computational time. Applying the same variance selection process used with SOAP did not provide improved results either.

For the PDDF, different discretizations were explored for a radius of 16 Å and a total of eight properties. Discretized with 0.8 Å bins and a gaussian spreading of 1 Å, thus resulting in 160 features, the PDDF already works in building a band gap model for all datasets—except the cubic perovskites—when scaled to standard variance. A finer discretization with 0.1 Å bins for the PDDF results in markedly improved results, while increasing the number of features 8-fold (1280). Using a simple, one-layer linear-activation autoencoder architecture trained on the [0, 1]-scaled PDDF representation of the training data alone, allows encoding the fingerprint into a 160-feature representation again. Using this representation with KRR consistently reaches the performance of the full representation hinting that the PDDF fingerprint indeed represents a low-dimensional manifold describing the data. Further studies could be conducted to explore whether and how the latent space is actually a representation of this manifold and how it relates to basic input structural data. In a similar vein, Schrier^65^ explored the eigenspectrum of the Coulomb matrix fingerprint for molecular data and found that even this already shrunken representation can be further reduced.

Finally, the GNN architecture and graph construction were implemented from Xie and Grossman,^23^ which proposed to use the same, manually tuned architecture for a wide array of problem sets. This seems valid, as a cursory screening of different graphing parameters and slightly modified neural network architectures did not result in any improvement.

Detailed results can be found in Table 2 for the band gap and the SI for energy predictions and the remaining SOAP and MBTR-related experiments.

## Results and Discussion

In refs (19, 46, 50, 51), both the PDDF approach and SOAP yield comparable prediction accuracy below 120 meV MAE with a slight lead for the SOAP fingerprint. In the case of the PDDF, both increasing the number of discretization steps and using the weighting proposed by Hemmer^66^ considerably improve results compared to the original rediscovered approach.^19^ In contrast, the results of the SOAP method seem relatively independent of parameterization in spherical and radial basis functions. Prediction is not changed by decreasing the smearing of the atomic positions (“+fine”-attribute), but for refs (19 and 50) increasing the radius expanded in the fingerprint results in a marked improval. The sine matrix approach is only significantly improving on the dummy predictions in the case of constant system size^46^ or with the hybrid perovskite dataset incorporating a large number of atoms for all systems.^51^ Except for ref (46), all MBTR parameterizations lag behind, regardless of the specific setup. The same holds true for the GNN, which consistently only reaches the performance of the “worst” fingerprinting method. While all best-performing prediction MAEs are of similar magnitude, it is notable that the baseline differs: in refs (46, 50, 51), the error of educated guessing is ≈800 meV, while it is only ≈300 meV in ref (19).

Conversely, in ref (49), the MBTR representation discretized on the inverse radius grid shows the best results, albeit model quality measured with the *R*^2^ coefficient does not reach the best results of the previously discussed datasets and the best MAE is nearly doubled to 250 meV. Interestingly, it is trailed only slightly by the GNN, while both SOAP and the PDDF are performing worse for this dataset.

Similarly, for the large-cell data from ref (36) the PDDF approach performs worst, independent of parameterization. With errors of 130 and 140 meV respectively, SOAP and MBTR are leading the fingerprint-based approaches, while the GNN is actually reaching the cherry-picked results from the original publication, which we could not reproduce with the SOAP fingerprint given. Compared to the other databases with comparable model MAEs, this is a considerably smaller improvement on random guessing!

Finally, for the cubic perovskites,^48^ no model reaches a satisfactory *R*^2^ even with the dataset reduced to nonmetallic compounds only. MBTR leads the field for fingerprint approaches with an MAE of 700 meV followed by SOAP and the PDDF in 50 meV increments. Again, the GNN shows the best result, improving by around 50 meV upon the best MBTR-based predictions, which is however still of a comparable magnitude and well within the margin of error of the MBTR-based approach. Here, the proposed methods for building a surrogate model seem to fail, possibly a result of the discontinuous nature of the input structures just being the results of simple combinatorics. Thus, for the sparse SOAP and MBTR fingerprints, most features just are incomparable with some parts being nonzero only in singular samples. In this case, the integrated approach of the GNN, dynamically building a fingerprint of the neighborhood based on properties alone seems to be at an advantage, even leading to a significantly improved *R*^2^ of 0.68 ± 0.08.

**Figure 3:**
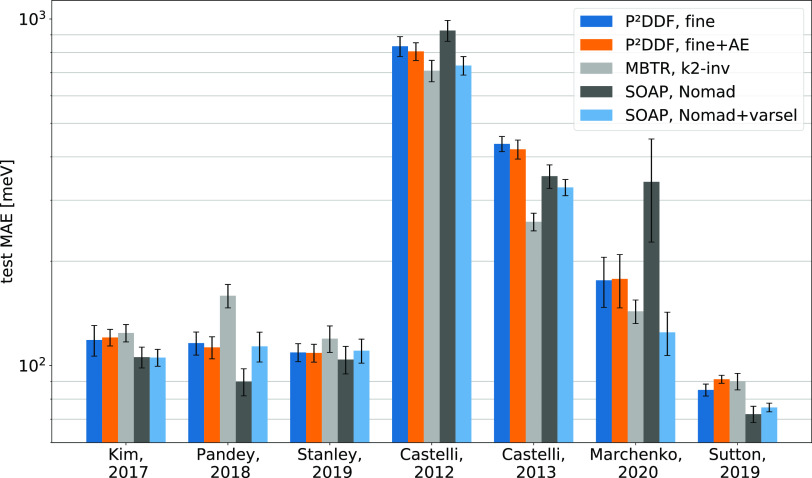
Band gap prediction error visualized for selected fingerprint models across all databases.

Overall, as visualized in Figure 3 and further shown for all parameterizations in the SI, the exact choice of specific fingerprinting parameters or even the basic method, as indicated by the inclusion of GNN-based results, has a much less pronounced effect on resulting errors than the choice of the database. Even for technically very pathological parameterizations, e.g., smoothing distributions with Gaussians of a similar width to the distribution range or the opposite for SOAP, the errors do not change on the order of magnitudes. While this study did not perform any large-scale fingerprint hyperparameter tuning^61^—instead choosing to replicate previous studies’ methodology, spanning a wide range of parameters—this indicates that for most practical screening applications, the choice of method is less important than having a “suitable” database. “Suitable” in this case goes far beyond the addition of new datapoints, as the failure of building a very good model for the data from refs (48 and 49) shows. While one might attribute this to the large amount of unique species in these datasets (see Table 1), a comparison between the similar results for the data from refs (19), (46), (50), and (51), shows that this is not the only deciding factor. This becomes especially apparent in the direct comparison of the databases from ref (51) and ref (50) where the number of available compounds is similar, yet the number of unique species is much higher in ref (50).

**Figure 4:**
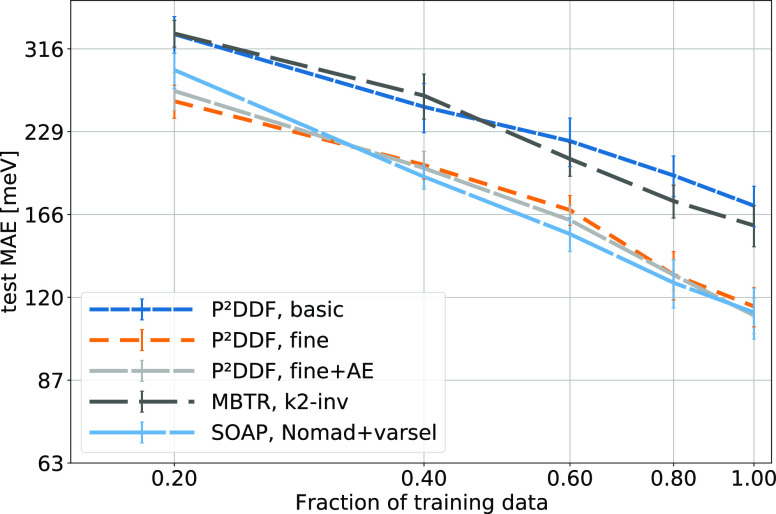
Learning curves for the data from ref (50). The test MAE for selected machine learning models is also shown.

**Figure 5:**
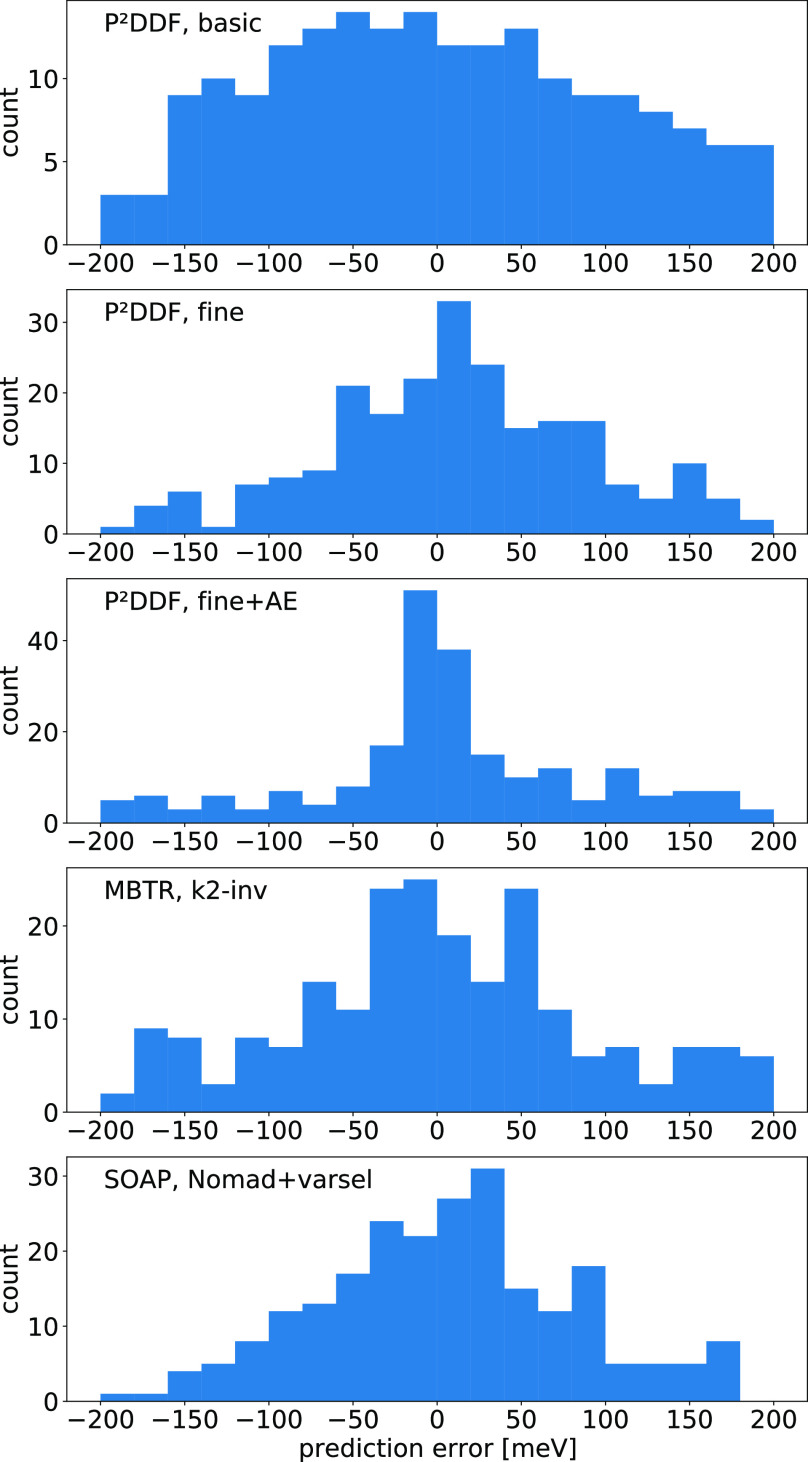
Error distribution for the best-performing models for the data from ref (50) for selected machine learning models.

To provide further insight into model quality and limitations, Figures 4 and 5 provide learning curves and error distributions for the band gap prediction on the data from ref (50). The curves plot the average MAE of models evaluated on a 20% test set versus the fraction of the respective training set used for creating the model. All fingerprint methods and feature extraction techniques show a consistent improvement with increasing training data with no sign of flattening out, indicating that more training data could be used to further improve model quality. In the low-data regime, MAEs are exceeding 250 meV and the models based on the autoencoded PDDF as well as the variance-selected SOAP significantly trail the pure, small-bin PDDF. By increasing the amount of data used for model creation, both SOAP and Autoencoder-model performance reach parity with usage of the full PDDF in model creation. The autoencoder results could be related to a biased sampling, which is not able to fully capture all structural features available in the dataset. In a real prediction setting, one might thus include a larger array of prospective compounds, including the finally predicted datapoints for training the autoencoder.

Checking the error distributions for different best-case results with an 80/20-train/test split shows gaussian-like distributions, so there is no inherent bias of any of the tested modeling procedures (compare Figure 5).

**Figure 6:**
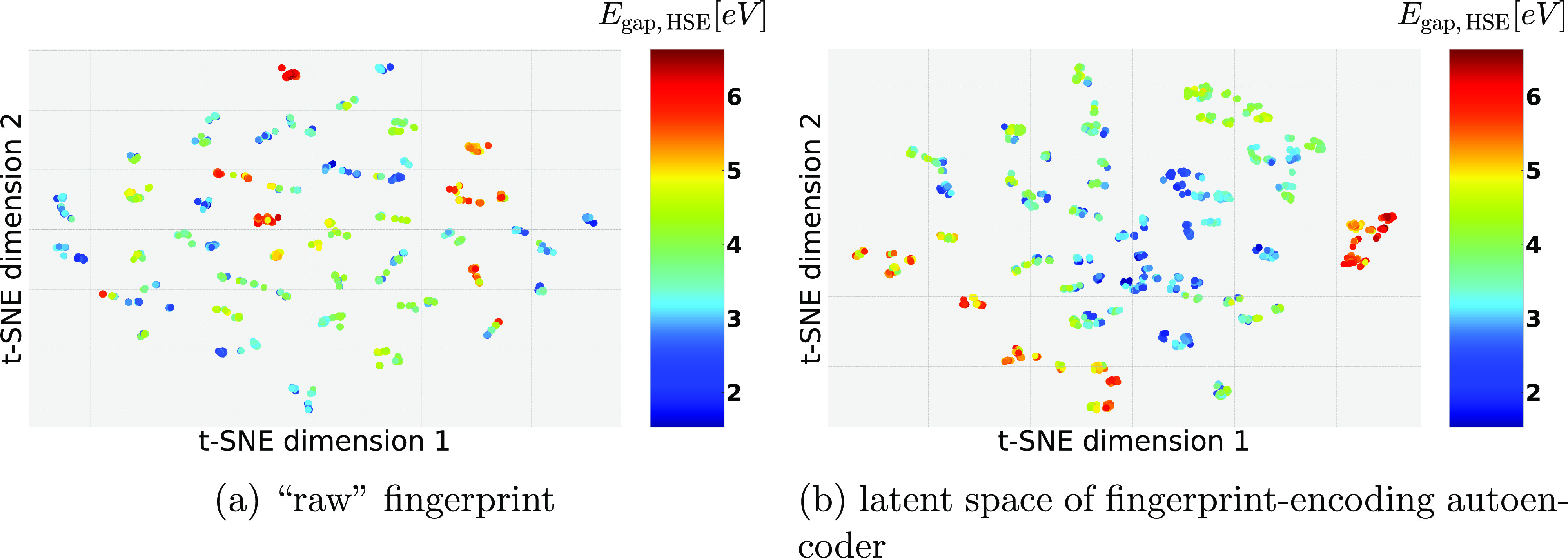
t-SNE reduced PDDF fingerprint and its encoding in 2D. Band gap overlayed and color-coded to relate to physical realities.

Additionally, in a first effort to understand the effects of the autoencoder on the PDDF fingerprint vectors used as input in the KRR model, t-SNE embeddings are used to create a two-dimensional map of the relative “neighborhoods” accessible in the fingerprint (see Figure 6).^67,68^ The dataset from ref (51) was used because it has a clear ABX_3_ perovskite structure and a relatively well working model, so a relation to physical quantities is relatively easy. When overlaying the band gap on a plot of the first two t-SNE dimensions, it is evident that the autoencoder preserves information about the physical characteristics of the system and the resulting models are no statistical artifact compared to using the PDDF. In the example, it even seems like the autoencoded representation is able to capture the band-gap-landscape in a much more continuous way than the original fingerprint, where a large number of singular high band-gap values are interspersed in the t-SNE-map. This observation can be related to the fact that the autoencoded representation clusters depending on A and X sites (see the SI for the t-SNE-plots for A-, B-, and X-site occupation), with the B site not clearly distinguishable as separate clusters in 2D. Conversely, the raw fingerprint does cluster mainly by the B and X occupation, while the molecular ions at the A site are not distinguishable in 2D clusters. As previous studies have shown that the B ion is not very relevant for the band gap,^19,69^ this hints that the autoencoder might actually be able to extract a “chemically informed” representation from the fingerprints. Obviously, the realizable advantage of this in building ML surrogates may be limited, as these are generally built on a space with a much higher dimension and the model can exploit more complicated relations than visualizable in a 2D map.

## Conclusions

The key finding of this study is that all currently competitive methods to create surrogate models for the prediction of materials properties are not able to capture arbitrary databases evenly yet. While a fraction of this might be attributed to varying complexity of the databases, the utter failure to capture a “good” band gap model in the conceptually very simple, large database of cubic perovskites^48^ hints that these methods in their commonly used form are not fit to replace DFT to model “discontinuous” relationships, where one just replaces a single atom with another compound-unique species (a finding evident already in a previous work^19^). However, for varying “alloys” and superstructures in a more or less continuous way, such as it happens in the other databases, as well as in Sutton et al.,^46^ the outlined methods seem to be able to perform quite well; an MAE of around 100 meV is great, comparing the inherent inaccuracies of experiments and DFT (GGA vs hybrids).^70^ For the latter databases, the GNN performs worse than all classic fingerprint approaches.

Additionally, for all studied descriptors, this study could not establish a strong, order-of-magnitude variation in per-dataset model performance for varying fingerprint parameters within the boundaries of previously published work, hinting that for all practical applications, a fine-grained hyperparameter search^61^ might be inefficient. Across all datasets, no method consistently reached the best performance, though SOAP is leading for several datasets. Setting aside different modeling techniques for the raw data, the available results for the band-gap models also indicate that choosing a method, much less choosing appropriate parameters for it, has much less influence than choosing a dataset. Thus, these findings question the significance of performing studies on isolated, proprietary datasets aiming for ever better numerical results without establishing baseline performance metrics and a comparison framework.^35−37^

From a technical perspective, the fact that fingerprinting functions creating input vectors of length several times the sample size work so well is quite unclear. Normally one would expect a strong overfitting to the test set, as the models have more free parameters than fitted samples. While that is exactly the reason for using a regularizing ML method, such as Kernel ridge regression (KRR), the high sparsity of both the SOAP and MBTR fingerprint for highly diverse databases could as well mean that the model only learns from a fraction of the supplied input data.^71^ The results of this study, which show SOAP with simple variance-based filtering of input features leading the field, underline this problem. This should warrant further investigation, as it also means that the given model will never be able to achieve full DFT accuracy just learning the substructure of, e.g., O, F, and N atoms, which incidentally are the shared building blocks of the cubic perovskite set, where MBTR excels but has accuracy in a range comparable to compounds swapping the A and B ions.^69^

It should also be noted that original authors open-sourcing their data or even publishing ML models should include a recommended training/test split so that results between methods can be compared across different publications.^20^ This is especially important, as the usage of neural networks in innovative ways slowly reaches the materials science field and thus the relatively simple and easily comparable fingerprinting approaches will be subject to an onslaught of “novel” approaches.^23,24,35^ To date and in light of this study, these seem to achieve the performance improvement desired by the community for a novel contribution mostly through careful data or target selection^h^ and profit from the intransparency of most documented uses of fingerprinting approaches.^36^ In the short term, averaging over multiple train/test splits and validating against established methodology seem a good stop-gap measure advocated also beyond this paper.^56,61^ As a long-term goal, the creation of larger, better verified datasets including all quantities of interest for the whole set and allowing us to break out large subsets of interesting structures is desirable. With the aim of the Materials Project and OQMD project,^57,74^ their current focus seems to lie on “verified” materials compared to a structured exploration of conformational space, which is necessary for effective surrogates. This might also be necessary to escape the fact that actual model performance is more tied to the data than to the model, which looks eerily similar to the state of natural-language processing 20 years ago.^75^

The availability of large-scale databases could also facilitate a more detailed examination of dimensionality reduction and its workings. While this study shows that the PDDF fingerprint seems to incorporate information on a low-dimensional manifold for the given datasets and this information in fact allows us to construct models of equivalent quality, it is not clear whether this approach can be further improved and yield extended insights. t-SNE-analysis hints that the encoding preserves “chemical information” while significantly reducing the feature size. Thus, it eases the systematical optimization of the resulting surrogate model in search of new compounds, but it is unclear whether it is thus possible for the model to actually relate to properties of physically realizable compounds.

## Tools and Data Availability

All calculations were done in a Python environment using the numpy, pandas, and ase packages for basic data manipulation and structure file handling; plotting was done with matplotlib. Machine learning procedures were used/implemented with sklearn, tensorflow, and (for the GNN) pytorch-geometric,^76^ while the fingerprints were generated with Dscribe(54) and our own implementation of the PDDF. Code to reproduce all numerical experiments is available upon request from the authors. This includes tools to convert the various proprietary formats, with which the data used are distributed by original authors, into a unified format.

## Supporting Information Available

Tabular overview of all fingerprint parameters for numerical experiments; MAEs, *R*^2^ scores, and RMSEs for the subpar-performing fingerprints on the band gap prediction and for all energy prediction models; additional experiments on the data from ref (50) are included to show that for the PDDF, published performance of the fine-grained approach can not be reached with single-property PDDFs alone (PDF)

## Footnotes

a This might also be correlated to the prevalence of Kernel ridge regression (KRR)-based methods,^28,45,46^ which scale badly with very large (>100k samples) databases, such as from the OQMD project.

b NOTE: even for this very simple fingerprint formalism, one could replace the δ-function with a more continuous, Gaussian-like one, add specific weights for specific neighbor atoms *i*, and add a cutoff function *f*_c_, which limits the range, where ρ > 0.

c The reader should be aware that this database is apparently being updated. Thus, the shown statistics only show a snapshot prior to publication of this paper (2020-07-28).

d See the github-repo of the DScribe-software (https://github.com/SINGROUP/dscribe/issues/44).

e These could be thought and optimized as hyperparameters of the whole “machine”—though this hinders general applicability and requires expensive remodeling for new data.

f Note that the dataset in ref (36) is proprietary and based on a subset of the available database, so the results are not directly comparable.

g This specific radius was chosen so it captures the environment of the maximum “whole cell” for most compounds (compared with the cell vector geometrical average and maximum unit-cell vector lengths in 1). Also, it could be discretized conveniently for numerical experiments as a multiple of 2.

h Xie and Grossman^23^ published their work shortly before the last public dump of the MP database,^57^ and while it works fair enough on the whole dump (≈80k entries), the results are only really good with the given subselection (≈47k). While the MP database has moved to deprecate entries, this started only in 2019^72^ and does, as well as the additions to the previous iteration, not account for the huge difference.^73^
