# Supplementary material for: Global Property Prediction: A Benchmark Study on Open-Source, Perovskite-like Datasets
Source: ACS Omega. 2021 May 3;6(19):12722–32. doi: 10.1021/acsomega.1c00991 (PMC8154242; doi:10.1021/acsomega.1c00991)
Supplement: Supplementary file 1 — ao1c00991_si_001.pdf [file ao1c00991_si_001.pdf]

Supporting Information for: Global property prediction: A benchmark study on open source, perovskite-like datasets

Felix Mayr and Alessio Gagliardi*

Department of Electrical and Computer Engineering, Technische Universität München, München, Germany

E-mail: alessio.gagliardi@tum.de

Contents

List of Figures **S1**

List of Tables **S1**

S1 Fingerprint description **S3**

S2 Fingerprint parameters **S5**

S2.1 Sine Matrix S5

S2.2 SOAP S5

S2.3 MBTR S6

S2.4 PDDF S7

S3 GNN-setup **S8**

S4 Additional experiments with the PDDF **S9**

S5 Additional Prediction Results **S10**

S5.1 Bandgap prediction S10

S5.2 Energy prediction S10

S5.2.1 Discussion of the energy prediction results S10

S6 Additional t-SNE-plots **S15**

References **S21**

List of Figures

S1 Bandgap prediction error visualized for different fingerprints across all databases.S14

S2 Energy prediction error visualized for different fingerprints across all selected databases. S19

S3 t-SNE reduced PDDF fingerprint and its encoding in 2D with ion-species of the ABX3-perovskite-structure overlayed S20

List of Tables

S1 SOAP-parametrizations used in this experiment with DScribe-parameter values S6

S2 MBTR-parametrizations used in this experiment with DScribe-parameter values S7

S3 PDDF parametrizations used in this experiment S8

S4 Results for predicting the calculated bandgaps for different methods, all results in meV for the mean-absolute error (MAE). S9

S5 Results for predicting the calculated bandgaps for different methods, all results in meV for the mean-absolute error (MAE). S11

S6 Results for predicting the calculated bandgaps for different methods, all results in meV for the root-mean-squared error (RMSE). S12
S7 Results for predicting the calculated bandgaps for different methods, given are the R-correlation coefficients of predictions to ground-truth. S13
S8 Overview of available/chosen energy quantities in the employed datasets. . . S15
S9 Results for predicting the calculated formation energies for different methods, all results in meV/atom for the mean-absolute error (MAE). S16
S10 Results for predicting the calculated formation energies for different methods, all results in meV/atom for the root-mean-squared error (RMSE). S17
S11 Results for predicting the calculated formation energies for different methods, given are the R-correlation coefficients of predictions to ground-truth. S18

S1 Fingerprint description

The following is a a description of the fingerprints employed in the associated study. For further information, the original papers should be consulted.

The sine matrix, derived from the molecule-only Coloumb-matrix for the description of crystals, includes a non-unique notion of structure into a numerical fingerprint vector. The basic idea is to collect all pairwise terms of the form (with Z being the atomic number and r_{ab} the distance from a to b)

$$\frac{Z_a Z_b}{r_{ab}^2} \tag{1}$$

for a given system $\mathcal{S}$ $(a, b \in \mathcal{S})$ of n-atoms in an $n \times n$-matrix (and some special self-interaction terms for $a = b$). The resulting matrix is hardly comparable for systems varying in size, though it works very well for modeling different conformations of the same system.[S1] Using the eigenvalues of the matrix faciliates comparison across systems, but considerably decreases the available information.[S2]

The other used descriptors are derived from a shared basis, where for a given atom j, the environment is described by the atomic density (see[S3,S4]) [1] :

$$\rho_j(\vec{r}) = \sum_i \delta(|\vec{r} - \vec{r}_i|) \tag{2}$$

Also, as this formalism is “atom-centered”, any derived, numerical fingerprint is atom-local first and it is necessary to transform it to a “global” fingerprint to be used for predicting system-total properties for systems of varying compositions. This transformation is done using special kernel-functions with kernel-based machine-learning-techniques[S4] or by plainly averaging the output over all atoms.[S5–S7] [found verbatim in the main text]

The Smooth Overlap of Atomic Positions (SOAP) fingerprint expresses the local environment with atom positions expanded as gaussian (eq. 2) in the form of a power spectrum

[1]NOTE: even for this very simple fingerprint formalism, one could replace the δ-function with a more continous, gaussian-like one, add specific weights for specific neighbor atoms i and add a cutoff-function f_c, which limits the range, where $\rho > 0$.

$\hat{p}$ of l spherical harmonics and n radial basis functions for each pairing of species (Z_1, Z_2) in the modeled data. The originally proposed form[S3] uses a covariance kernel K to be used for comparison and in kernel-based statistical learning techniques modeling single-atom local environments. Further "meta"-kernels have beend defined,[S4] which combine the evaluation of subsequent kernel evaluations for all pairs of atoms in both structures either by simple averaging or by only restricting the average to "best matches" (REMatch-kernel). Necessitating a large number of kernel-evaluations, these approaches have been generally superseded by averaging of the individual atoms power-spectra in recent work.[S5,S6]

Historically, also radial distribution functions (RDF) have been used for describing a given atomic density in spherical shells from a center atom outward, with angular distribution functions (ADF) describing the spatial relation of the neighbors. This approach has been picked up and extended in the molecule focused FCHL-fingerprint,[S8] the Many-Body-Tensor-Representation (which generalizes to crystals) – proposed in a preprint[S9] - as well as the Property-Density-Distribution Function (PDDF).[S7]

The MBTR approach consists of 3 distinct parts k: the first part ($k = 1$) builds a fingerprint vector by discretizing a range spanning the number of species in the data, where a gaussian function is placed on each "number", corresponding to a species in the fingerprinted structure. The second part ($k = 2$) is similar to a partial radial distribution function, collecting averaged RDFs (with a gaussian spreading) for each species-tuple (Z_1, Z_2) within a defined cutoff-radius. Innovating compared to a "classic" partial RDF, the distribution can be discretized along the inverse distance. The $k = 3$-term is subsequently discretizing an angular distribution function (for each angle formed by the triple species (Z_1, Z_2, Z_3)) either on a cosine or angle-based grid. Depending on the grids used in discretization, the resulting "flat" fingerprint vector for a database with a high number of species n, easily reaches a length of several thousand parts: for $k = 2$ alone: $(\sum_i^n i) \times \text{gridsize})$ - where for any individual sample, the vector is very sparse.

Contrary, in the PDDF, the fingerprint size is fixed by appropriate discretization of a

property-weighted RDF averaged across all atoms N in the system:[S7]

$$\mathrm{PDDF}(r) = \frac{1}{N}\sum_{j}^{N}\sum_{i,|r_i-r_j|<R_c} w_{\mathrm{property}} \exp\left(\frac{|r-(r_i-r_j)|}{\sigma^2}\right) \tag{3}$$

with the summation going over all atoms i in the periodic system within a specified cutoff-radius R_c. Updating the approach, the fingerprint vector now discretizes gaussian distributions of width σ instead of the delta-functions to be more continous. Bins can be further normalized by either the number of atoms in a spherical-shell or the size of the spherical shell itself. This approach has been proposed in application to molecules previously by Hemmer.[S10] He also proposes another property-weighting function w using the product of the property of the center atom p_α and the respective environment atom p_β $w_{\mathrm{prod}} = p_\alpha \cdot p_\beta$ instead of just $w_{\mathrm{original}} = p_\beta$.

S2 Fingerprint parameters

Note: parameters not mentioned were kept at the defaults of the respective library.

S2.1 Sine Matrix

The sine matrix fingerprints were created using the DScribe-library[S5] (git commit-id: `90aa6c4b1397a223f1330e8bb93bbb24726e8078`). To get a comparable fingerprint for all compounds in a database varying in size, the eigenspectrum of the sine-matrix was chosen as a fingerprint.

S2.2 SOAP

Periodic SOAP fingerprints were also created using the aforementioned DScribe-library. For each model, only the compounds available in the database were used for creating fingerprint elements with the `gto`-type radial basis functions. To get a global fingerprint, `outer`

averaging was employed.

Specifically, for the "codenames" used in the text, the parameters were varied as shown in table S1.

Table S1: SOAP-parametrizations used in this experiment with DScribe-parameter values

	`rcut`	`nmax`	`lmax`	`sigma`
De	6	4	8	1.0
Marchenko	6	8	6	1.0
Marchenko, LR	16	8	6	1.0
Nomad	10	4	4	0.5
Nomad, fine	10	4	4	0.1

S2.3 MBTR

Basic parameters for the MBTR fingerprints (again using the DScribe-implementation) were taken from the authors publications[S9,S11] (SI available on request from the authors due to the preprint status). The later publication does extensive hyperparameter tuning to find the best model, which is not necessarily good in a benchmark setting, thus parameters where handpicked within the range given in the paper. A parameter not tuned in these studies is the number of bins n, when discretizing the different distributions used in the fingerprint. Choosing $n = 100$ creates extremely large ($\mathcal{O}(100000)$) fingerprints with the used datasets, thus we decided to mainly use $n = 16$ (to keep in line with the PDDF cutoff discretization) along a 16Å cutoff-radius. Only k2 and k3-components were evaluated, with the shared fingerprint simply concatenated.

Throughout, the exponential weigthing functions taken from the example included in DScribe were employed. As the weighting is constant for each bin, in a standardizing ML-workflow, it should not matter, which function is chosen here. However, performance with scaling applied before training the ML-model was actually worse than without, so this parameter might have further influence.

To enable comparability of different perodic systems, the `'n_atoms'`-normalization was

selected. All tried parametrizations and their denominations can be found in table S2.

Table S2: MBTR-parametrizations used in this experiment with DScribe-parameter values

	k2/k3/k1	(min,max,n)	sigma	weighting (`exp`)
k2-inv	`inverse_distance/-`	($\frac{1}{16}$, 1, 16) / -	0.05/-	`scale=1.0, cutoff=1e-3`
k2-rdf	`distance`	(0.1, 16, 16)/-	1.0/-	`scale=0.75, cutoff=1e-2`
k2-inv-broad	`inverse_distance/-`	($\frac{1}{16}$, 1, 16)/ -	1/-	`scale=1.0, cutoff=1e-3`
k2-rdf-broad	`distance`	(0.1, 16, 16)/-	4/-	`scale=0.75, cutoff=1e-2`
k2-inv-100	`inverse_distance/-`	($\frac{1}{16}$, 1, 100)/ -	0.05/-	`scale=1.0, cutoff=1e-3`
k2-rdf-100	`distance`	(0.1, 16, 100)/-	1.0/-	`scale=0.75, cutoff=1e-2`
full	`inverse_distance/angle/atomic_number`	($\frac{1}{16}$, 1, 16)/(0, 180, 10)/(1, no. of species, no. of species)	0.05/5/1	`scale=0.75, cutoff=1e-3/ scale=0.5, cutoff=1e-3/` none

S2.4 PDDF

The Property-Density-Distribution-Function PDDF was used as outlined in[S7] with a per-bin-normalization equivalent to the volume of the spherical shell (which is irrelevant when pre-scaling before building the ML model as well). Additionally, each "distribution" was smoothed with a gaussian of width σ and the approach of Hemmer[S10] to weight by the product of center- and "outer"-atom property was added. Properties were used as tabulated by the MENDELEEV-package.

Both second and first ionization energies, the atomic radius, the electronegativity, the electron affinity and the number of electrons in total and in the p & s valence shell were used in the given order for construction of the total PDDF.

Specifically the parameter shown in table S3 were selected.

Table S3: PDDF parametrizations used in this experiment

	weighting	r_{cut}[Å]	binsize	σ
PDDF, basic	plain	16	0.8	1
P^2DDF, basic	product	16	0.8	1
PDDF, fine	plain	16	0.1	1
P^2DDF, fine	product	16	0.1	1
PDDF, fine/sharp	plain	16	0.1	0.2
P^2DDF, fine/sharp	product	16	0.1	0.2

S3 GNN-setup

For the Graph-Neural-Network (GNN) experiments, the code published by Xie and Grossman[S12] (at github.com/txie-93/cgcnn) was modified to work with the PYTORCH-GEOMETRIC framework.[S13]

The graph structures were created from atomic structures by using the provided one-hot-encoded atomic properties as the node-feature. The graph connectivity matrix was built using connections from each atom to its first 12 neighbors within a radius of 10 ∘A in the periodic structure. The edge property vectors encode the distance between connected nodes by evaluating a gaussian function centered at the respective distance on a $[0, 10]$-grid with a spacing of 0.2.

These graphs were used in a supervised learning regression scenario being the feature-input to the GNN from Xie including Graph-Convolutional-Network (GCN) layers. The original setup of the GNN was used under the term "GNN, Xie", using 4 GCN-layers processing a node embeding of 64 units, which was reduced after pooling through 2 fully connected layers ($64 \rightarrow 32 \rightarrow 1$) to output the model target. Since not a lot of justification is given for this architecture in the paper, we also tried a computationally lighter setup "GNN, small-Xie", using just 3 GCN-layers processing a node embeding of 48 units, which after pooling was again reduced through 2 fully connected layers ($48 \rightarrow 16 \rightarrow 1$) to output the model target. Performance was within the margin of error of the original implementation with a slight edge for the latter. Training was done for 800 epochs with a batch size of 256 all throughout. The network was optimized with a standard SGD-optimizer (starting

learning rate 0.01, momentum 0.9), where the learning rate was continously updated when plateauing (measured by a 80-20-train-validation-split)

Unfortunately, the energy evaluation for the data from[S7] didn't converge with this approach.

S4 Additional experiments with the PDDF

To illustrate that the given performance with the PDDF is not an artifact of a singular property, a separate study was conducted on the dataset from.[S14] Basic parameters correspond to the "P²DDF, fine"-setting, all single feature properties and various combinations were explored and results can be found in Table S4.

Table S4: Results for predicting the calculated bandgaps for different methods, all results in meV for the mean-absolute error (MAE).

	MAE [meV]	RMSE [meV]	R^2
cov_r_prod	485±13	638±16	-0.02±0.11
ion_1_prod	477±28	631±32	0.23±0.15
ion_2_prod	528±22	682±30	-0.12±0.17
r_prod	555±19	716±22	-0.44±0.14
val_p_prod	388±12	515±19	0.57±0.02
val_s_prod	578±24	731±27	-0.80±0.25
vdw_r_prod	582±22	746±23	-0.71±0.16
e_prod	522±22	656±30	0.03±0.13
ea_prod	386±21	528±31	0.54±0.06
en_prod	413±27	566±40	0.48±0.07
en+ea_prod	228±15	324±22	0.86±0.02
e+r_prod	471±18	601±11	0.28±0.08
val_p+val_s_prod	419±16	526±13	0.51±0.06
ion_2+ion_1_prod	429±19	552±23	0.43±0.12
ion_2+ion_1+en+ea_prod	154±9	232±20	0.93±0.01
en+ea+e+val_p+val_s_prod	138±13	220±16	0.94±0.01
ion_2+ion_1+en+ea+val_p+val_s_prod	126±9	188±11	0.95±0.01
ion_2+ion_1+en+ea+val_p+val_s+e+r_prod	116±9	184±13	0.96±0.01

S5 Additional Prediction Results

S5.1 Bandgap prediction

To supplement the key results in the main paper, all evaluated MAEs are shown in Table S5 and the corresponding RMSEs and R2-scores in Table S6 and Table S7, respectively. In Figure S1, for each "class" of used fingerprinting functions, errors are visualized for all databases used. It is clearly evident, that non-pathological parameters do not change results a lot for a given dataset.

S5.2 Energy prediction

Similar to the bandgap prediction in the main paper, one can predict global energy properties. As the energy is an extensive quantity in DFT, per-atom-normalized targets have to be chosen chosen. Also, not every database includes energies readily available. While it is possible to build models for the "total DFT-energy/atom", these are relatively inaccurate and thus not considered in the evaluation. Specifically the quantities as outlined in Table S8 were chosen as the energy target.

All evaluated MAEs are shown in Table S9 and the corresponding RMSEs and R2-scores in Table S10 and Table S11, respectively. In Figure S2, for each "class" of used fingerprinting functions, errors are visualized for all databases used.

S5.2.1 Discussion of the energy prediction results

The energy predictions for both[S15] and[S7] lie in the $\approx 2-4\frac{\text{meV}}{\text{atom}}$ range, while for,[S6] results are considerably improved up to $\approx 1.1\frac{\text{meV}}{\text{atom}}$. Note that the latter results are a considerable improvement upon the work published with the dataset, even when adjusting for the "per cation"-results published therein.[S6] It should be noted though that therein the modeling efficacy was evaluated by the "total" performance of a given fingerprint/modeling-combination on a single test-set, while herein, the best models are chosen for a single objective. It's

Table S5: Results for predicting the calculated bandgaps for different methods, all results in meV for the mean-absolute error (MAE).

	Kim[S15]	Pandey[S14]	Stan.[S7]	Cas.[S16]	Cas.[S17]	Mar.[S18]	Sutton[S6]
handpicked	381±11	-	-	-	-	-	-
dummy	884±34	730±19	323±23	1270±73	1530±46	332±15	845±16
sinematrix, eigenspectrum	368±15	538±39	212±15	1088±77	1102±60	298±22	141±8
GNN, Xie	185±13	154±10	130±18	655±71	262±21	107±9	92±9
GNN, small-Xie	192±14	156±18	137±18	670±53	266±20	113±11	99±4
PDDF, basic	172±11	199±13	134±11	930±80	551±16	179±11	101±4
PDDF, fine	141±8	139±14	114±12	888±57	481±19	176±20	90±4
PDDF, fine+AE	142±6	143±7	110±12	879±61	490±28	170±19	91±4
P^2DDF, basic	159±12	172±14	136±19	888±69	521±19	207±32	96±3
P^2DDF, fine	118±12	116±9	109±7	834±55	436±22	176±29	85±3
P^2DDF, fine+AE	120±7	113±8	109±6	806±48	421±27	178±31	91±2
MBTR, k2-inv	124±7	159±12	120±11	709±50	260±15	143±11	90±5
MBTR, k2-rdf	128±7	144±13	126±10	786±57	305±18	140±18	93±6
MBTR, k2-inv-100	124±6	160±13	120±11	705±52	262±16	145±11	89±5
MBTR, k2-rdf-100	127±8	146±14	125±10	787±59	301±17	146±14	95±6
MBTR, k2-inv-broad	171±11	283±8	131±13	867±62	362±19	149±15	125±7
MBTR, k2-rdf-broad	159±10	228±23	133±12	875±60	345±18	148±13	112±7
MBTR, full	124±7	158±13	120±11	709±52	260±16	141±12	89±5
MBTR, k2-inv+varsel	125±8	178±12	126±14	768±63	348±27	142±15	86±4
MBTR, k2-rdf+varsel	149±10	164±9	154±13	835±67	394±22	139±13	95±5
MBTR, k2-inv-broad+varsel	178±12	286±12	133±11	967±81	435±25	132±22	127±11
MBTR, k2-rdf-broad+varsel	156±10	255±18	138±14	926±39	422±37	127±19	116±10
MBTR, k2-inv-100+varsel	125±7	177±12	124±15	766±59	350±26	151±18	88±5
MBTR, k2-rdf-100+varsel	154±9	168±9	153±9	822±49	392±25	151±26	99±5
MBTR, full+varsel	112±8	130±8	140±21	771±44	331±25	148±22	89±6
SOAP, Marchenko	100±8	85±9	109±7	1067±75	349±27	494±90	70±5
SOAP, De	107±6	97±9	108±8	1071±74	329±25	442±95	78±4
SOAP, Nomad	106±7	90±8	104±10	926±64	352±27	339±112	72±4
SOAP, Nomad, fine	195±11	281±8	181±13	823±68	443±23	144±17	149±8
SOAP, Marchenko, LR	110±11	96±7	122±10	1288±130	645±58	939±330	75±3
SOAP, Marchenko+varsel	101±6	111±10	123±8	738±52	309±24	132±17	77±6
SOAP, De+varsel	106±9	112±10	116±7	777±50	339±23	135±20	78±4
SOAP, Nomad+varsel	105±6	114±11	110±9	734±45	327±18	125±18	76±2
SOAP, Nomad, fine+varsel	106±6	117±10	110±8	731±48	316±17	146±36	77±4
SOAP, Marchenko, LR+varsel	99±8	90±5	104±8	745±48	324±27	129±25	76±3

Table S6: Results for predicting the calculated bandgaps for different methods, all results in meV for the root-mean-squared error (RMSE).

	Kim[S15]	Pandey[S14]	Stan.[S7]	Cas.[S16]	Cas.[S17]	Mar.[S18]	Sutton[S6]
handpicked	446±8	-	-	-	-	-	-
dummy	1073±40	887±19	429±32	1580±114	1870±48	414±23	1020±26
sinematrix, eigenspectrum	485±19	681±50	293±24	1396±89	1467±111	398±64	232±18
GNN, Xie	252±20	225±16	180±31	879±83	446±49	148±17	150±13
GNN, small-Xie	255±20	223±25	188±31	907±78	447±49	155±17	157±10
PDDF, basic	227±19	278±21	196±28	1199±89	800±29	269±53	166±9
PDDF, fine	192±8	209±15	159±23	1151±68	709±36	287±60	151±19
PDDF, fine+AE	190±9	216±12	153±22	1125±66	729±37	267±62	150±8
P^2DDF, basic	210±16	248±22	200±34	1154±89	786±32	356±110	159±7
P^2DDF, fine	175±18	184±13	144±9	1084±51	664±45	307±94	142±14
P^2DDF, fine+AE	171±11	183±15	144±11	1044±52	642±44	308±109	146±9
MBTR, k2-inv	171±12	239±32	159±15	917±66	461±43	226±56	150±14
MBTR, k2-rdf	173±11	233±38	167±15	1032±62	512±38	197±52	154±12
MBTR, k2-inv-100	171±12	240±34	159±16	913±64	466±47	217±45	150±11
MBTR, k2-rdf-100	173±15	234±36	164±16	1030±62	506±33	235±69	158±13
MBTR, k2-inv-broad	232±25	404±13	170±16	1135±64	586±36	236±65	200±20
MBTR, k2-rdf-broad	214±16	350±30	176±18	1152±64	566±31	229±58	182±15
MBTR, full	170±12	238±33	159±15	917±69	461±44	223±59	147±12
MBTR, k2-inv+varsel	177±12	265±28	171±19	1064±214	584±69	247±80	145±8
MBTR, k2-rdf+varsel	200±14	248±17	208±19	1146±229	641±76	221±65	155±10
MBTR, k2-inv-broad+varsel	247±30	410±27	171±13	1413±185	697±121	210±89	211±33
MBTR, k2-rdf-broad+varsel	216±19	379±27	188±22	1249±83	663±89	196±71	189±17
MBTR, k2-inv-100+varsel	177±11	258±17	166±21	1016±110	583±71	313±70	154±35
MBTR, k2-rdf-100+varsel	206±14	261±14	206±16	1072±76	627±61	260±108	156±9
MBTR, full+varsel	168±23	231±28	219±101	1018±79	561±56	301±92	143±10
SOAP, Marchenko	151±14	158±20	150±11	1713±230	744±78	1431±724	121±13
SOAP, De	148±10	177±24	143±17	1676±222	639±59	1265±637	129±10
SOAP, Nomad	149±12	168±28	140±15	1333±136	553±26	1066±727	121±11
SOAP, Nomad, fine	247±14	385±10	251±20	1070±76	631±35	216±43	230±16
SOAP, Marchenko, LR	181±20	194±27	165±13	2229±393	1805±208	2929±1978	130±9
SOAP, Marchenko+varsel	146±11	206±33	161±11	946±58	528±51	252±89	132±32
SOAP, De+varsel	158±13	203±16	152±11	1001±62	558±52	253±81	130±12
SOAP, Nomad+varsel	151±10	203±38	145±11	944±57	540±48	247±89	130±10
SOAP, Nomad, fine+varsel	152±13	207±32	143±12	944±66	521±42	303±147	135±17
SOAP, Marchenko, LR+varsel	146±13	174±18	138±13	948±60	577±83	253±98	130±10

Table S7: Results for predicting the calculated bandgaps for different methods, given are the R-correlation coefficients of predictions to ground-truth.

	Kim[S15]	Pandey[S14]	Stan.[S7]	Cas.[S16]	Cas.[S17]	Mar.[S18]	Sutton[S6]
handpicked	0.79±0.01	-	-	-	-	-	-
dummy	-	-	-	-	-	-	-
sinematrix, eigenspectrum	0.75±0.03	-0.01±0.33	0.09±0.18	-2.21±0.73	0.04±0.15	-3.78±2.06	0.95±0.01
GNN, Xie	0.94±0.01	0.94±0.01	0.81±0.07	0.68±0.08	0.94±0.01	0.87±0.03	0.98±0.00
GNN, small-Xie	0.94±0.01	0.94±0.02	0.80±0.07	0.66±0.09	0.94±0.01	0.86±0.03	0.98±0.00
PDDF, basic	0.95±0.01	0.89±0.02	0.72±0.08	-0.06±0.25	0.80±0.02	0.55±0.10	0.97±0.00
PDDF, fine	0.97±0.00	0.94±0.01	0.82±0.05	0.15±0.16	0.84±0.02	0.53±0.13	0.98±0.01
PDDF, fine+AE	0.97±0.00	0.94±0.01	0.83±0.06	0.17±0.15	0.83±0.02	0.58±0.14	0.98±0.00
P^2DDF, basic	0.96±0.01	0.92±0.02	0.69±0.12	0.06±0.20	0.80±0.03	0.35±0.22	0.98±0.00
P^2DDF, fine	0.97±0.01	0.96±0.01	0.86±0.03	0.24±0.13	0.86±0.02	0.49±0.21	0.98±0.00
P^2DDF, fine+AE	0.97±0.00	0.96±0.01	0.85±0.03	0.36±0.07	0.87±0.02	0.50±0.21	0.98±0.00
MBTR, k2-inv	0.97±0.00	0.92±0.02	0.83±0.05	0.50±0.09	0.94±0.01	0.67±0.11	0.98±0.00
MBTR, k2-rdf	0.97±0.00	0.93±0.02	0.81±0.05	0.30±0.09	0.92±0.01	0.71±0.14	0.98±0.00
MBTR, k2-inv-100	0.97±0.00	0.92±0.02	0.82±0.05	0.49±0.09	0.94±0.01	0.67±0.09	0.98±0.00
MBTR, k2-rdf-100	0.97±0.00	0.93±0.02	0.81±0.06	0.30±0.10	0.92±0.01	0.65±0.14	0.98±0.00
MBTR, k2-inv-broad	0.95±0.01	0.75±0.03	0.79±0.07	-0.05±0.13	0.90±0.02	0.64±0.15	0.96±0.01
MBTR, k2-rdf-broad	0.96±0.01	0.84±0.03	0.77±0.07	-0.08±0.14	0.91±0.01	0.65±0.15	0.97±0.01
MBTR, full	0.97±0.00	0.92±0.02	0.83±0.05	0.50±0.09	0.94±0.01	0.67±0.12	0.98±0.00
MBTR, k2-inv+varsel	0.97±0.00	0.91±0.02	0.79±0.06	0.40±0.11	0.89±0.02	0.65±0.13	0.98±0.00
MBTR, k2-rdf+varsel	0.96±0.00	0.92±0.01	0.65±0.05	0.33±0.11	0.87±0.03	0.71±0.11	0.98±0.00
MBTR, k2-inv-broad+varsel	0.95±0.01	0.74±0.04	0.79±0.05	-0.07±0.23	0.85±0.05	0.74±0.16	0.96±0.01
MBTR, k2-rdf-broad+varsel	0.96±0.01	0.80±0.04	0.75±0.07	0.14±0.12	0.86±0.04	0.75±0.15	0.96±0.01
MBTR, k2-inv-100+varsel	0.97±0.00	0.91±0.01	0.80±0.05	0.40±0.11	0.89±0.02	0.55±0.14	0.98±0.01
MBTR, k2-rdf-100+varsel	0.96±0.00	0.91±0.01	0.66±0.06	0.36±0.12	0.87±0.03	0.63±0.19	0.98±0.00
MBTR, full+varsel	0.97±0.01	0.93±0.02	0.67±0.23	0.47±0.11	0.90±0.02	0.59±0.15	0.98±0.00
SOAP, Marchenko	0.98±0.00	0.97±0.01	0.86±0.02	0.29±0.11	0.86±0.03	0.11±0.14	0.99±0.00
SOAP, De	0.98±0.00	0.96±0.01	0.87±0.04	0.29±0.11	0.89±0.02	0.15±0.13	0.98±0.00
SOAP, Nomad	0.98±0.00	0.96±0.01	0.87±0.03	0.33±0.12	0.91±0.01	0.23±0.23	0.99±0.00
SOAP, Nomad, fine	0.94±0.01	0.76±0.03	0.38±0.12	0.25±0.19	0.87±0.02	0.70±0.09	0.94±0.01
SOAP, Marchenko, LR	0.97±0.01	0.95±0.01	0.84±0.03	0.18±0.10	0.49±0.07	0.04±0.07	0.98±0.00
SOAP, Marchenko+varsel	0.98±0.00	0.94±0.02	0.82±0.03	0.50±0.07	0.91±0.02	0.67±0.18	0.98±0.01
SOAP, De+varsel	0.98±0.00	0.95±0.01	0.84±0.03	0.36±0.09	0.90±0.02	0.67±0.13	0.98±0.00
SOAP, Nomad+varsel	0.98±0.00	0.95±0.02	0.85±0.02	0.43±0.11	0.91±0.02	0.68±0.16	0.98±0.00
SOAP, Nomad, fine+varsel	0.98±0.00	0.94±0.02	0.86±0.03	0.47±0.10	0.91±0.01	0.58±0.28	0.98±0.00
SOAP, Marchenko, LR+varsel	0.98±0.00	0.96±0.01	0.86±0.03	0.48±0.06	0.90±0.03	0.67±0.18	0.98±0.00

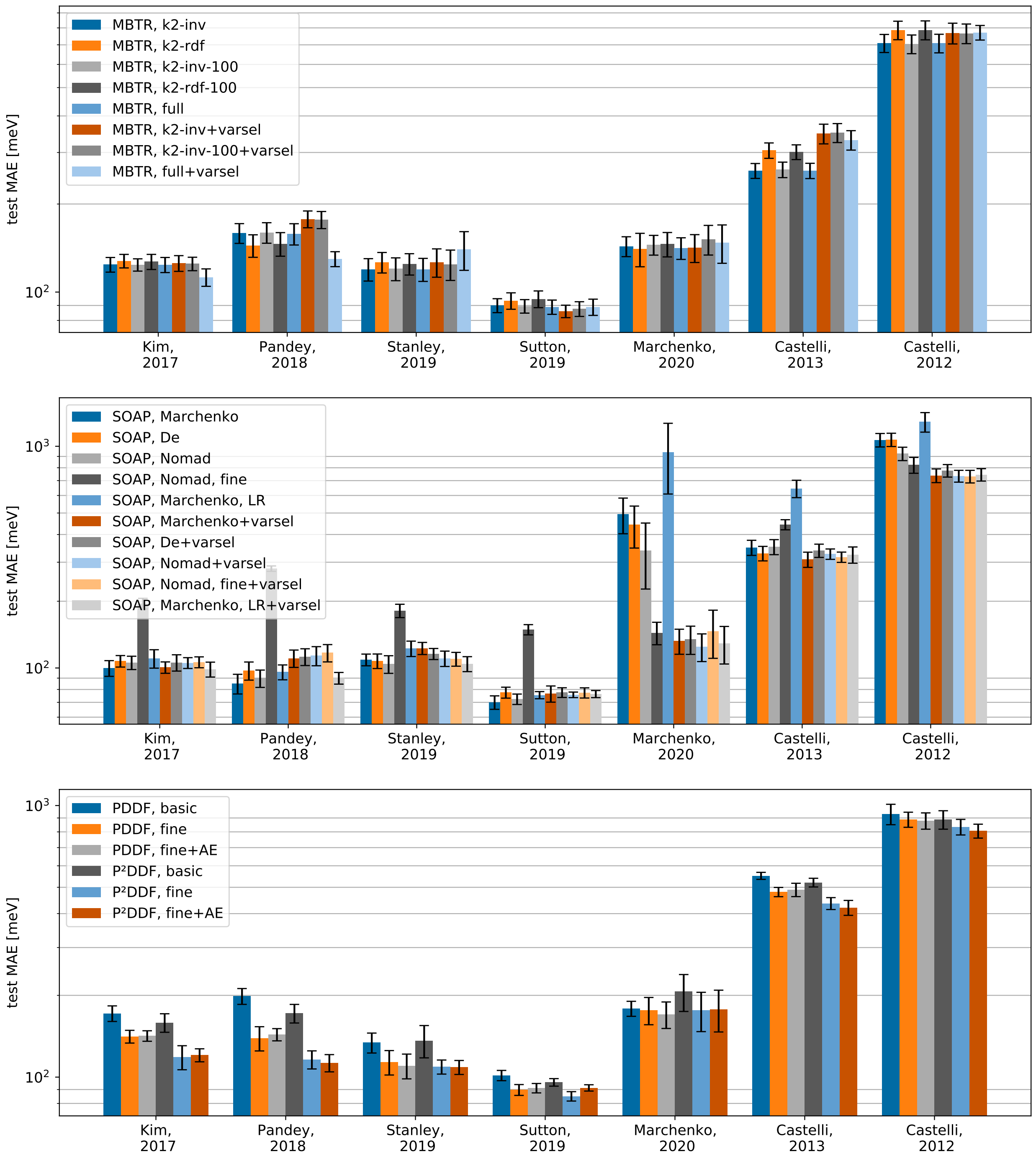

Figure S1: Bandgap prediction error visualized for different fingerprints across all databases.

Table S8: Overview of available/chosen energy quantities in the employed datasets.

Kim[S15]	atomization/formation energy: $E_{\mathrm{ABX_3}} - \sum_i n_i E_i$, where $E_{\mathrm{ABX_3}}$ is the DFT calculated total energy and n_i/E_i are count and energy of the isolated atoms, normalized per atom
Pandey[S14]	energies available with respect to the most stable structure. A large portion of available structures are 0, e.g. stable. No regression model was built.
Stanley[S7]	atomization/formation energy, normalized per atom
Castelli[S16]	includes a heat of formation quantity in DB, given unit is eV, but it doesn't line up with the provided sample. No regression model was built.
Castelli[S17]	only a total energy available. No regression model was built.
Marchenko[S18]	no energy property available
Sutton[S6]	formation energy **per atom** (in the competition paper, results are *per cation*)

notable, how the R^2-score indicates near perfect models for[S15] and.[S6]

Using the average number of atoms in each database, the best given MAEs would result in formation energy errors for the final compound [2] of $\approx 0.7\frac{\mathrm{kcal}}{\mathrm{mol}}$,[S15] $\approx 1.2\frac{\mathrm{kcal}}{\mathrm{mol}}$[S7] and $\approx 1.5\frac{\mathrm{kcal}}{\mathrm{mol}}$,[S6] respectively. This is on the order of what is reported as chemical accuracy in.[S19]

S6 Additional t-SNE-plots

Figure S3 shows the t-SNE-data from the main publication overlayed with the different "characteristic" ions of an ABX3-perovskite, as taken from.[S15]

[2] not that this is not per "unit"-cell, normalized vs. a basic compound

Table S9: Results for predicting the calculated formation energies for different methods, all results in meV/atom for the mean-absolute error (MAE).

	Kim[S15]	Sutton[S6]	Stan.[S7]
handpicked	109.1±5.9	-	-
dummy	262.0±10.1	34.3±1.0	119.1±11.7
sinematrix, eigenspectrum	70.7±3.7	10.5±0.3	38.8±7.4
GNN, Xie	9.0±1.3	3.2±0.3	(n.c)
GNN, small-Xie	8.5±1.1	3.2±0.4	(n.c.)
PDDF, basic	4.8±0.3	6.3±0.3	4.5±0.5
PDDF, fine	3.5±0.7	4.4±0.3	3.0±0.5
PDDF, fine+AE	4.2±0.3	4.6±0.3	3.8±0.3
P^2DDF, basic	3.9±0.3	5.9±0.3	5.6±0.7
P^2DDF, fine	2.7±0.3	3.7±0.2	3.6±0.9
P^2DDF, fine+AE	3.8±0.5	4.3±0.3	4.4±0.8
MBTR, k2-inv	2.1±0.3	2.0±0.1	2.7±0.3
MBTR, k2-rdf	2.6±0.3	2.5±0.1	2.7±0.2
MBTR, k2-inv-100	2.0±0.3	1.9±0.2	2.6±0.2
MBTR, k2-rdf-100	2.8±0.3	2.2±0.1	2.6±0.2
MBTR, k2-inv-broad	3.0±0.3	5.4±0.4	3.6±0.4
MBTR, k2-rdf-broad	3.9±1.0	6.1±0.3	3.1±0.4
MBTR, full	2.0±0.2	1.9±0.1	2.7±0.3
MBTR, k2-inv+varsel	2.4±0.2	2.0±0.1	3.1±0.6
MBTR, k2-rdf+varsel	5.0±0.3	2.8±0.1	4.0±0.5
MBTR, k2-inv-broad+varsel	4.8±0.8	5.8±0.3	3.4±0.3
MBTR, k2-rdf-broad+varsel	5.6±0.5	5.3±0.2	3.1±0.4
MBTR, k2-inv-100+varsel	2.6±0.2	2.0±0.1	4.0±0.6
MBTR, k2-rdf-100+varsel	5.7±0.4	3.0±0.1	4.5±0.7
MBTR, full+varsel	4.0±0.3	2.0±0.1	10.3±7.9
SOAP, Marchenko	6.9±0.6	0.7±0.1	4.0±0.4
SOAP, De	5.3±0.7	0.7±0.1	2.5±0.4
SOAP, Nomad	3.8±0.4	0.7±0.0	2.6±0.2
SOAP, Nomad, fine	8.5±0.4	12.8±0.2	3.7±0.6
SOAP, Marchenko, LR	10.1±1.1	1.4±0.1	7.0±0.7
SOAP, Marchenko+varsel	3.4±0.4	1.4±0.1	7.0±0.6
SOAP, De+varsel	4.9±0.5	1.6±0.1	4.5±0.5
SOAP, Nomad+varsel	2.5±0.2	1.1±0.1	4.6±0.6
SOAP, Nomad, fine+varsel	2.5±0.2	1.2±0.1	4.4±0.5
SOAP, Marchenko, LR+varsel	4.5±0.4	1.5±0.1	7.7±0.9

Table S10: Results for predicting the calculated formation energies for different methods, all results in meV/atom for the root-mean-squared error (RMSE).

	Kim[S15]	Sutton[S6]	Stan.[S7]
handpicked	160.3±10.1	-	-
dummy	346.7±11.3	41.9±1.2	147.5±13.5
sinematrix, eigenspectrum	103.6±7.2	17.2±1.3	84.1±42.0
GNN, Xie	13.7±2.5	5.4±0.5	(n.c.)
GNN, small-Xie	12.6±2.3	5.4±0.7	(n.c)
PDDF, basic	7.6±1.0	10.6±1.0	5.9±0.7
PDDF, fine	5.5±0.9	7.6±0.7	4.1±1.0
PDDF, fine+AE	6.2±0.5	7.8±0.7	5.1±0.7
P^2DDF, basic	5.7±0.5	9.9±1.2	7.7±1.1
P^2DDF, fine	3.9±0.6	6.5±1.0	5.1±1.6
P^2DDF, fine+AE	5.7±1.0	7.4±0.8	5.9±1.3
MBTR, k2-inv	3.2±0.3	3.9±1.3	4.6±0.9
MBTR, k2-rdf	4.0±0.5	4.6±0.9	4.3±1.0
MBTR, k2-inv-100	3.2±0.4	3.6±1.1	4.5±1.0
MBTR, k2-rdf-100	4.1±0.5	4.0±0.4	4.3±1.0
MBTR, k2-inv-broad	4.5±0.4	8.7±2.0	5.1±1.1
MBTR, k2-rdf-broad	5.9±1.4	9.6±2.2	4.5±0.9
MBTR, full	3.1±0.2	3.7±1.2	4.6±0.9
MBTR, k2-inv+varsel	4.2±0.5	3.6±0.8	4.7±1.5
MBTR, k2-rdf+varsel	7.7±0.8	4.6±0.6	5.8±1.1
MBTR, k2-inv-broad+varsel	6.9±1.0	9.1±1.4	4.8±0.7
MBTR, k2-rdf-broad+varsel	8.7±2.3	8.4±0.9	4.5±1.0
MBTR, k2-inv-100+varsel	4.3±0.6	3.6±0.5	6.0±1.9
MBTR, k2-rdf-100+varsel	8.4±0.6	4.9±0.6	6.4±1.6
MBTR, full+varsel	6.2±0.8	3.3±0.3	29.3±42.7
SOAP, Marchenko	12.5±1.7	1.5±0.4	5.3±0.6
SOAP, De	9.9±1.3	1.3±0.5	3.5±0.9
SOAP, Nomad	7.1±1.0	1.4±0.5	3.5±0.5
SOAP, Nomad, fine	12.6±0.8	17.0±0.4	5.1±1.2
SOAP, Marchenko, LR	19.9±3.0	3.2±0.4	9.9±1.6
SOAP, Marchenko+varsel	5.7±0.7	2.7±0.7	9.6±1.8
SOAP, De+varsel	7.8±1.1	3.3±0.7	6.2±0.9
SOAP, Nomad+varsel	4.1±0.4	2.4±0.8	6.3±1.0
SOAP, Nomad, fine+varsel	4.1±0.5	2.2±0.4	5.9±0.6
SOAP, Marchenko, LR+varsel	7.1±0.7	2.9±0.7	11.0±2.0

Table S11: Results for predicting the calculated formation energies for different methods, given are the R-correlation coefficients of predictions to ground-truth.

	Kim[S15]	Sutton[S6]	Stan.[S7]
handpicked	0.72±0.05	-	-
dummy	-	-	-
sinematrix, eigenspectrum	0.90±0.01	0.80±0.04	0.73±0.17
GNN, Xie	1.00±0.00	0.98±0.00	(n.c.)
GNN, small-Xie	1.00±0.00	0.98±0.00	(n.c.)
PDDF, basic	1.00±0.00	0.93±0.01	1.00±0.00
PDDF, fine	1.00±0.00	0.97±0.01	1.00±0.00
PDDF, fine+AE	1.00±0.00	0.96±0.01	1.00±0.00
P^2DDF, basic	1.00±0.00	0.94±0.01	1.00±0.00
P^2DDF, fine	1.00±0.00	0.97±0.01	1.00±0.00
P^2DDF, fine+AE	1.00±0.00	0.97±0.01	1.00±0.00
MBTR, k2-inv	1.00±0.00	0.99±0.01	1.00±0.00
MBTR, k2-rdf	1.00±0.00	0.99±0.01	1.00±0.00
MBTR, k2-inv-100	1.00±0.00	0.99±0.01	1.00±0.00
MBTR, k2-rdf-100	1.00±0.00	0.99±0.00	1.00±0.00
MBTR, k2-inv-broad	1.00±0.00	0.95±0.03	1.00±0.00
MBTR, k2-rdf-broad	1.00±0.00	0.94±0.03	1.00±0.00
MBTR, full	1.00±0.00	0.99±0.01	1.00±0.00
MBTR, k2-inv+varsel	1.00±0.00	0.99±0.00	1.00±0.00
MBTR, k2-rdf+varsel	1.00±0.00	0.99±0.00	1.00±0.00
MBTR, k2-inv-broad+varsel	1.00±0.00	0.95±0.02	1.00±0.00
MBTR, k2-rdf-broad+varsel	1.00±0.00	0.96±0.01	1.00±0.00
MBTR, k2-inv-100+varsel	1.00±0.00	0.99±0.00	1.00±0.00
MBTR, k2-rdf-100+varsel	1.00±0.00	0.99±0.00	1.00±0.00
MBTR, full+varsel	1.00±0.00	0.99±0.00	0.93±0.13
SOAP, Marchenko	1.00±0.00	1.00±0.00	1.00±0.00
SOAP, De	1.00±0.00	1.00±0.00	1.00±0.00
SOAP, Nomad	1.00±0.00	1.00±0.00	1.00±0.00
SOAP, Nomad, fine	1.00±0.00	0.75±0.02	1.00±0.00
SOAP, Marchenko, LR	1.00±0.00	0.99±0.00	1.00±0.00
SOAP, Marchenko+varsel	1.00±0.00	1.00±0.00	1.00±0.00
SOAP, De+varsel	1.00±0.00	0.99±0.00	1.00±0.00
SOAP, Nomad+varsel	1.00±0.00	1.00±0.00	1.00±0.00
SOAP, Nomad, fine+varsel	1.00±0.00	1.00±0.00	1.00±0.00
SOAP, Marchenko, LR+varsel	1.00±0.00	1.00±0.00	0.99±0.00

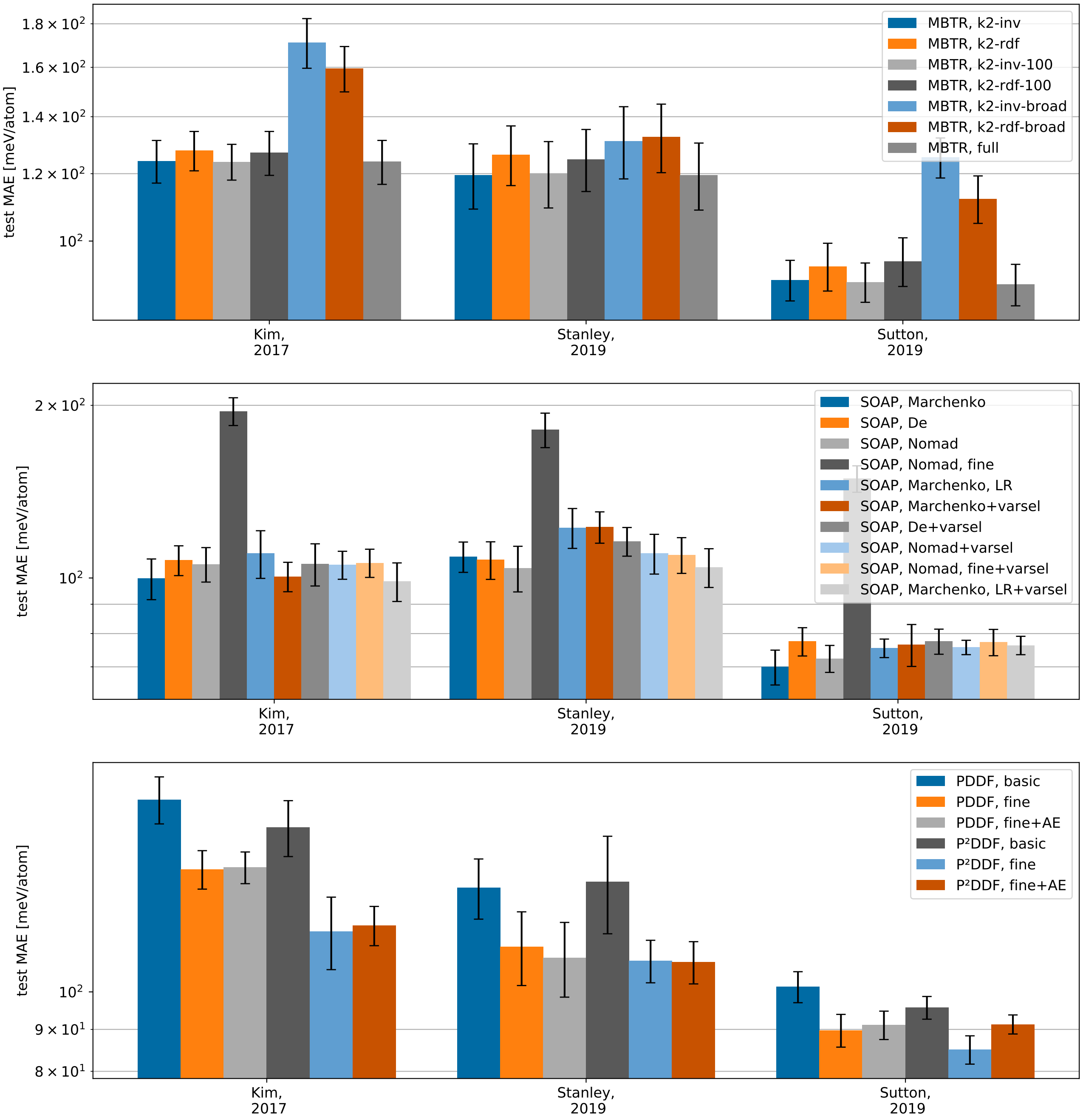

Figure S2: Energy prediction error visualized for different fingerprints across all selected databases.

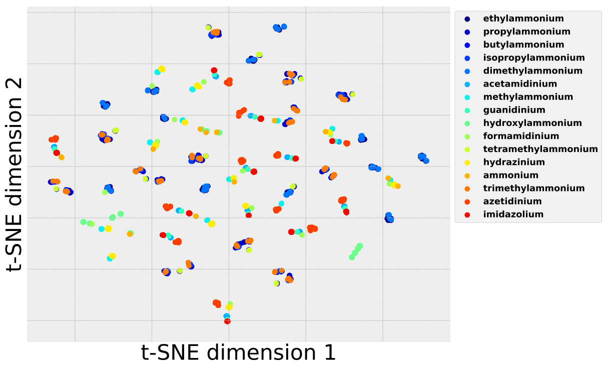

(a) A-ion ("raw" fingerprint)

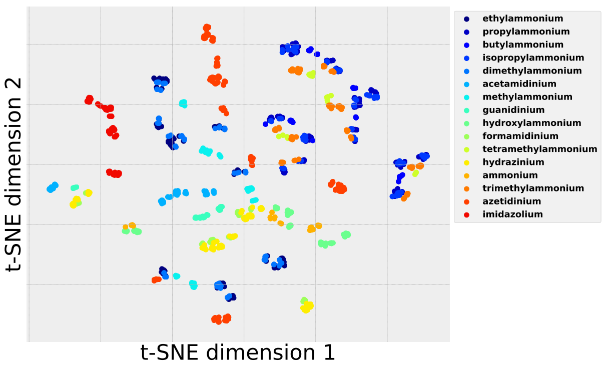

(b) A-ion (latent space)

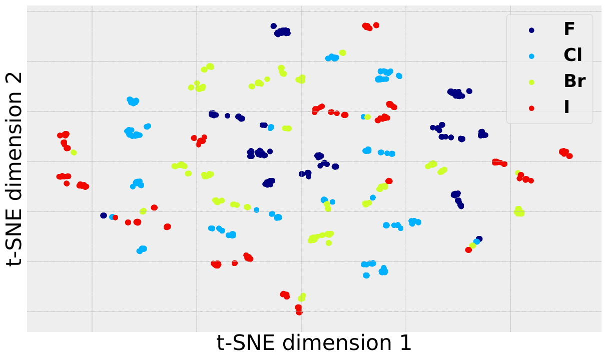

(c) B-ion ("raw" fingerprint)

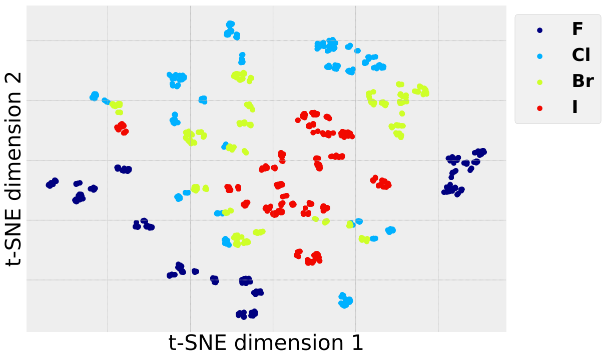

(d) B-ion (latent space)

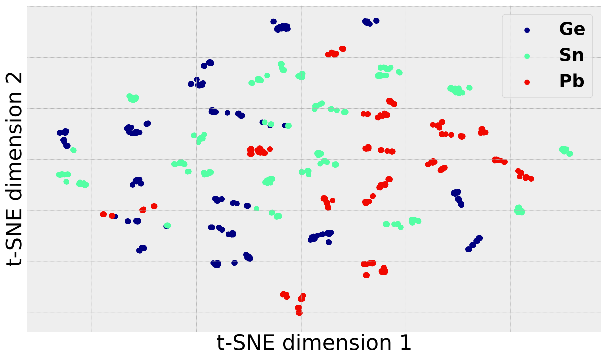

(e) X-ion ("raw" fingerprint)

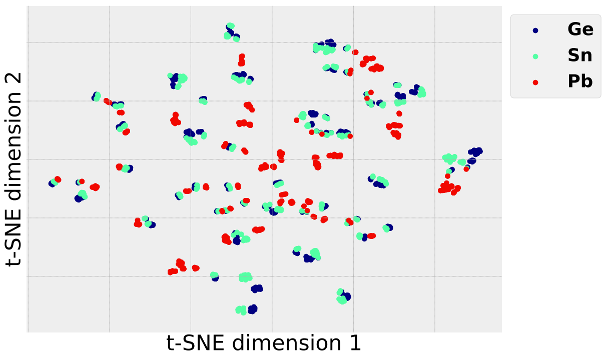

(f) X-ion (latent space)

Figure S3: t-SNE reduced PDDF fingerprint and its encoding in 2D with ion-species of the ABX3-perovskite-structure overlayed

References

[S1] Rinderle, M.; Kaiser, W.; Mattoni, A.; Gagliardi, A. Machine-Learned Charge Transfer Integrals for Multiscale Simulations in Organic Thin Films. *J. Phys. Chem. C* **2020**, *124*, 17733–17743.

[S2] Rupp, M.; Tkatchenko, A.; Müller, K.-R.; von Lilienfeld, O. A. Fast and Accurate Modeling of Molecular Atomization Energies with Machine Learning. *Phys. Rev. Lett.* **2012**, *108*.

[S3] Bartók, A. P.; Kondor, R.; Csányi, G. On representing chemical environments. *Phys. Rev. B* **2013**, *87*.

[S4] De, S.; Bartók, A. P.; Csányi, G.; Ceriotti, M. Comparing molecules and solids across structural and alchemical space. *Phys. Chem. Chem. Phys.* **2016**, *18*, 13754–13769.

[S5] Himanen, L.; Jäger, M. O.; Morooka, E. V.; Canova, F. F.; Ranawat, Y. S.; Gao, D. Z.; Rinke, P.; Foster, A. S. DScribe: Library of descriptors for machine learning in materials science. *Comput. Phys. Commun.* **2020**, *247*, 106949.

[S6] Sutton, C.; Ghiringhelli, L. M.; Yamamoto, T.; Lysogorskiy, Y.; Blumenthal, L.; Hammerschmidt, T.; Golebiowski, J. R.; Liu, X.; Ziletti, A.; Scheffler, M. Crowd-sourcing materials-science challenges with the NOMAD 2018 Kaggle competition. *npj Comput. Mater.* **2019**, *5*.

[S7] Stanley, J. C.; Mayr, F.; Gagliardi, A. Machine Learning Stability and Bandgaps of Lead-Free Perovskites for Photovoltaics. *Adv. Theory Simul.* **2019**, *3*, 1900178.

[S8] Faber, F. A.; Christensen, A. S.; Huang, B.; von Lilienfeld, O. A. Alchemical and structural distribution based representation for universal quantum machine learning. *J. Chem. Phys.* **2018**, *148*, 241717.

[S9] Huo, H.; Rupp, M. Unified Representation of Molecules and Crystals for Machine Learning.

[S10] Hemmer, M. C. Radial Distribution Functions in Computational Chemistry - Theory and Applications. Ph.D. thesis, 2007.

[S11] Langer, M. F.; Goeßmann, A.; Rupp, M. Representations of molecules and materials for interpolation of quantum-mechanical simulations via machine learning. 2020.

[S12] Xie, T.; Grossman, J. C. Crystal Graph Convolutional Neural Networks for an Accurate and Interpretable Prediction of Material Properties. *Phys. Rev. Lett.* **2018**, *120*.

[S13] Fey, M.; Lenssen, J. E. Fast Graph Representation Learning with PyTorch Geometric. ICLR Workshop on Representation Learning on Graphs and Manifolds. 2019.

[S14] Pandey, M.; Jacobsen, K. W. Promising quaternary chalcogenides as high-band-gap semiconductors for tandem photoelectrochemical water splitting devices: A computational screening approach. *Phys. Rev. Mater.* **2018**, *2*.

[S15] Kim, C.; Huan, T. D.; Krishnan, S.; Ramprasad, R. A hybrid organic-inorganic perovskite dataset. *Sci. Data* **2017**, *4*, 170057.

[S16] Castelli, I. E.; Landis, D. D.; Thygesen, K. S.; Dahl, S.; Chorkendorff, I.; Jaramillo, T. F.; Jacobsen, K. W. New cubic perovskites for one- and two-photon water splitting using the computational materials repository. *Energy Environ. Sci.* **2012**, *5*, 9034.

[S17] Castelli, I. E.; García-Lastra, J. M.; Hüser, F.; Thygesen, K. S.; Jacobsen, K. W. Stability and bandgaps of layered perovskites for one- and two-photon water splitting. *New J. Phys.* **2013**, *15*, 105026.

[S18] Marchenko, E. I.; Fateev, S. A.; Petrov, A. A.; Korolev, V. V.; Mitrofanov, A.; Petrov, A. V.; Goodilin, E. A.; Tarasov, A. B. Database of 2D hybrid perovskite

materials: open-access collection of crystal structures, band gaps and atomic partial charges predicted by machine learning. *Chem. Mater.* **2020**,

[S19] Faber, F. A.; Hutchison, L.; Huang, B.; Gilmer, J.; Schoenholz, S. S.; Dahl, G. E.; Vinyals, O.; Kearnes, S.; Riley, P. F.; von Lilienfeld, O. A. Prediction Errors of Molecular Machine Learning Models Lower than Hybrid DFT Error. *J. Chem. Theory Comput.* **2017**, *13*, 5255–5264.
